# From school climate to brain development: What the ABCD study reveals about the educational context of adolescence^☆^

**DOI:** 10.1016/j.dcn.2026.101746

**Published:** 2026-05-25

**Authors:** Erin L. Thompson, Oliver M. Sawi, Ethan A. Roy, Shermaine Abad, Christine M. Kaiver, Sarah M. Lehman, Jolene Tay, Marybel R. Gonzalez, Amandine Van Rinsveld, Gayathri J. Dowling, Sandra A. Brown, Terry L. Jernigan, Bruce D. McCandliss, Elizabeth A. Hoffman

**Affiliations:** a Center for Children and Families, Florida International University, USA; b Graduate School of Education, Stanford University, USA; c J. Craig Venter Institute, USA; d Laureate Institute for Brain Research, USA; e Department of Psychiatry and Behavioral Health, The Ohio State University, USA; f The National Institute on Drug Abuse, The National Institutes of Health, USA; g Department of Psychiatry, UC San Diego, USA; h Center for Human Development and Cognitive Science Department, UC San Diego, USA; i The National Institute on Drug Abuse, The National Institutes of Health, USA

**Keywords:** Educational neuroscience, School context, Academic achievement, Adolescent development, ABCD study

## Abstract

Children and adolescents spend much of their time in school, and educational environments are central to neurodevelopment and socioemotional health. However, educational constructs are often underrepresented in developmental cognitive neuroscience research. The Adolescent Brain Cognitive Development^SM^ (ABCD) Study provides an unprecedented opportunity to examine schooling within a large, diverse, longitudinal cohort with rich neuroimaging and socioenvironmental data. This narrative review synthesizes education-related ABCD publications identified through a comprehensive search of the ABCD publication repository. Across 109 studies (representing approximately 7% of ABCD Study publications), educational environments emerged as multilevel systems that shape development through interconnected pathways. Supportive school environments were modestly associated with more favorable neurodevelopment, emotional and behavioral functioning, and daily health-related behaviors, often buffering the effects of prior child adversity. In contrast, adverse school experiences, including discipline, unfair treatment, and school lockdowns, functioned as meaningful sources of stress that contributed to mental health concerns, earlier substance use initiation, and physical health risk, particularly when occurring alongside stress in other settings. In several cases, these relationships were bidirectional, suggesting that youth behavior and school experiences may reinforce one another over time. Overall, ABCD findings indicate that school environments are not merely background contexts or proxies for socioeconomic conditions, but distinct and modifiable factors that can both exacerbate risk and promote resilience. Future research leveraging longitudinal, multilevel, and externally linked contextual data will be critical for identifying when and how educational environments can be targeted to improve adolescent outcomes.

## Highlights

• 109 articles examined education-related constructs within the ABCD Study, including 40 articles examining neurodevelopment.
• Findings suggest bidirectional associations between educational environments and adolescent development.
• Limited research has focused on school- and district-level characteristics, including academic achievement data.

## Graphical Abstract

**Figure:**
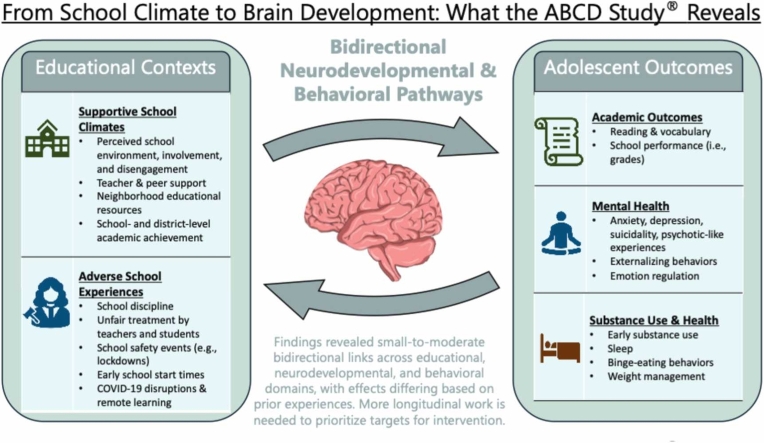

## 1 Introduction

Children and adolescents spend a significant amount of their waking hours in school, positioning learning environments as a critical context for neuroplasticity throughout childhood, adolescence, and into early adulthood (Amran et al., 2019, Hoferichter and Raufelder, 2025; McCandliss, 2010). Schooling also provides structured, sustained experiences that shape academic learning, social development, and health behaviors. Research increasingly suggests that these environments are both contributors to and outcomes of neurodevelopment (e.g., Roy et al., 2024, Sheridan et al., 2025, Sheridan and McLaughlin, 2016). Despite this, educational constructs remain underrepresented in developmental cognitive neuroscience research (Leisman, 2022). Therefore, to accurately characterize typical or atypical developmental trajectories, researchers must consider school contexts alongside family, community, and other ecological contexts, as key correlates of developmental processes.

### 1.1 Conceptual frameworks for studying educational environments

Two complementary research frameworks provide a foundation for understanding how school environments shape adolescent development: educational neuroscience and bioecological theory. Educational neuroscience provides a framework for understanding how learning environments shape brain development through experience-dependent neuroplasticity (Amran et al., 2019, Hoferichter and Raufelder, 2025; Leisman, 2022; McCandliss, 2010). This perspective emphasizes that schooling is not simply a setting in which cognitive abilities are expressed, but rather a context that actively contributes to the development of neural systems supporting complex skills such as reading, language, and mathematics. Importantly, this framework also recognizes bidirectional processes, in which individual differences in neurocognitive functioning shape how youth engage with learning environments, which, in turn, may reinforce or modify neural development (Hoferichter and Raufelder, 2025). From this perspective, variation in school quality, instructional opportunities, and academic engagement can have measurable effects on neurodevelopment, underscoring the importance of studying educational environments as active contributors to developmental change.

The Bioecological Model of Human Development (Bronfenbrenner and Morris, 2006) situates school environments within larger social and environmental systems. This model conceptualizes development as resulting from ongoing interactions between individuals and multiple layers of environmental influence, including immediate settings such as family and school (microsystems), connections between settings (mesosystems), and broader influences (exosystems and macrosystems). Schools both function within and reflect these broader systems, shaping access to opportunities and exposure to risk. School experiences may mirror, amplify, or buffer broader socioeconomic inequality, neighborhood conditions, and policy environments on development. By emphasizing these multilevel and dynamic processes, bioecological theory provides a foundation for understanding how school environments interact with other contexts to influence developmental trajectories. Together, these frameworks highlight schooling not merely as an academic setting or a proxy for socioeconomic status, but as a distinct developmental environment with measurable neurobiological, behavioral, and contextual consequences that can range from promotive to prohibitive.

### 1.2 The ABCD study as a platform for studying educational environments

The Adolescent Brain Cognitive Development^SM^ (ABCD) Study (https://abcdstudy.org/) offers unprecedented opportunities to better understand the broader environments youth experience, including educational contexts. Launched in 2016, the ABCD Study® enrolled nearly 12,000 U.S. children aged 9–10 years, following them longitudinally into early adulthood. ABCD recruitment was conducted primarily through public, public-charter, and private schools selected to approximate the national demographic distribution of U.S. 9–10-year-olds, although some deviations occurred, particularly with respect to socioeconomic status and urbanicity (Garavan et al., 2018, Gard et al., 2023). Participants complete comprehensive assessments of physical and mental health, substance use, family, community, and school environments, neurocognition, biospecimens, as well as structural and functional neuroimaging. These features make the ABCD Study uniquely suited to test conceptual models of how educational environments shape and are shaped by development across multiple levels of analysis.

### 1.3 Multilevel, multi–informant education data in the ABCD Study

A key strength of the ABCD Study is that it provides the type of multilevel, multimethod data required to operationalize educational neuroscience and bioecological theory in a prospective, longitudinal framework. The study incorporates education-relevant data from multiple informants (youth, caregivers, teachers) and links participant data to external data sources that characterize the environments in which participants learn, play, and live. These features enable fine-grained analyses of learning environments, academic trajectories, and school-based supports. Table 1 summarizes the education-related constructs available in the ABCD Study 6.1 public data release available on the NIH Brain Development Cohorts Data Hub (see https://www.nbdc-datahub.org/). This includes data collected from the main ABCD Study cohort, the Social Development (SD) Substudy, and the COVID-19 Rapid Response Research (RRR) Surveys. By bringing these measures together in a single location, the table provides a practical resource for researchers interested in examining the full range of education-related constructs in the ABCD Study.

**Table 1:** Comprehensive List of School-Related Measures in the ABCD Study 6.1 Data Release.

**Measure**	**Description**	**Citation**	**Youth**	**Caregiver**	**Teacher**	**Linked External Data**
**Nesting Variables**						
Pseudo School ID	Anonymized school ID released for participants whose school(s) had an NCES School ID	—	—	—	—	B–Y6
Pseudo District ID	Anonymized district ID whose district(s) had an NCES District ID	—	—	—	—	B–Y6
**School Characteristics**						
School Modality	Past-week school-at-home indicator	—	—	Y2–Y4	—	—
School Type (KSADS Background)	Public, private, charter, home school enrollment	Townsend et al., (2020)	—	B–Y6	—	—
Grade Level	Caregiver-reported school grade	Barch et al., (2021)	—	B–Y6	—	—
Grade Retention (KSADS Background)	Whether youth repeated a grade, and when	Townsend et al., (2020)	B–Y3	Y3, Y5	—	—
School/District Demographics (SEDA)	Size, disability, IEP, FRL, race/ethnicity	Fahle et al., (2021)	—	—	—	B
**Learning (Reading & Math)**						
NIH Toolbox Picture Vocabulary	Verbal intellect and vocabulary	Gershon et al., (2014); Thompson et al., (2019)	B, Y2, Y4, Y6	—	—	—
NIH Toolbox Oral Reading	Single-word reading accuracy	Carlozzi et al., (2015); Thompson et al., (2019)	B, Y2, Y4, Y6	—	—	—
Reading for Pleasure	Whether and how frequently youth read for fun	Sun et al., (2024)	Y3–Y6	B, Y1–Y4	—	—
SMARTE Math Tasks	Three computerized math tasks	Guillaume et al., (2024)	Y3, Y5	—	—	—
HOME-SF Learning Environment	Reading, books, puzzles at ages 3–5	Sugland et al., (1995)	—	5Y	—	—
**Academic Achievement**						
School Grades	Letter grades (A+–F)	Gonzalez et al., (2021)	Y2–Y3	Y2–Y6	—	—
School Grades ((KSADS Background)	Typical grades and grade changes	Townsend et al., (2020)	B–Y3	B–Y3	—	—
School Services (KSADS Background)	Special services received at school	Townsend et al., (2020)	—	B–Y6	—	—
IEP Participation	Whether youth has an IEP	Gonzalez et al., (2021)	—	Y2–Y6	—	—
Child Opportunity Index (Education Subdomain)	Education opportunity at census tract level	Acevedo-Garcia et al., (2020)	—	—	—	B
SEDA Achievement	Third-grade district-level math & reading intercept and slope over time	Fahle et al., (2021)	—	—	—	B
**Perceived School Climate & Support**						
SRPF School Environment	School support, relationships, opportunities	Gonzalez et al., (2021); Hamilton et al., (2011)	B–Y4, Y6	—	—	—
SRPF School Involvement	Participation in school activities	Gonzalez et al., (2021)	B–Y5	—	—	—
SRPF School Disengagement	Boredom and lack of interest in school	Gonzalez et al., (2021)	B–Y6	—	—	—
**School-Related Behaviors**						
Attendance	Excused and unexcused school absences	Gonzalez et al., (2021)	Y2–Y3	Y2–Y6	—	—
Caregiver School Support (MNBS)	Homework help and ensuring attendance	Kantor et al., (2004)	Y3, Y6	—	—	—
Peer School-Related Behavior	Friends skipping school, suspensions, grades	Bingham et al., (1995)	Y2–Y6	—	—	—
Teacher BPM	Teacher-reported internalizing, externalizing, and attention concerns	Achenbach et al., (2011)	—	—	B–Y6	—
School Start Time (MCTQ)	School start time	Zavada et al., (2005)	Y2–Y6	—	—	—
Sleep Schedule (MCTQ)	School vs weekend sleep	Zavada et al., (2005)	Y2–Y6	—	—	—
Fitbit Actigraphy Sleep	School vs weekend sleep onset, offset, duration	Haghayegh et al., (2019)	Y2, Y4, Y6	—	—	—
Fitbit Actigraphy Physical Activity	School vs weekend steps, METs	Haghayegh et al., (2019)	Y2, Y4, Y6	—	—	—
**School Experiences**						
KSADS School Discipline (KSADS Background)	Detention or suspension in past 12 months	Townsend et al., (2020)	B–Y4	B–Y6	—	—
Social Development Substudy Discipline	Sends home, detentions, suspensions, expulsions; skipping; cheating	Brislin et al., (2024)	SD W1–W6	SD W1–W6	—	—
School Lockdown	Exposure to lockdown events and reaction	Jackson, (2019)	Y3–Y6	Y3–Y6	—	—
School-Related Unfair Treatment	Discrimination from teachers and students	Phinney et al., (1998)	Y1, Y2, Y4, Y6	—	—	—
Changing Schools	Whether youth changed schools, and impact	Jackson, (2019)	B–Y6	B–Y6	—	—
School Behavior	Theft, property damage, aggression toward teachers/students	Brislin, Clark et al., (2024)	SD W1–W5	SD W1–W5	—	—
JVQ School Victimization	Bomb threat, theft, assault, bullying, vandalism	Finkelhor et al., (2005)	SD W1–W5	SD W1–W5	—	—
JVQ School Staff Awareness	Whether school adults are aware of victimization	Finkelhor et al., (2005)	SD W1–W5	SD W1–W5	—	—
Caregiver School Involvement (APQ)	PTA meetings, conferences, involvement	Frick et al., (1999)	SD W1–W5	SD W1–W5	—	—
School-Related Conflict (KSADS Background)	Grades as a source of caregiver–youth conflict	Townsend et al., (2020)	—	B–Y6	—	—
**COVID-19 School Disruptions (Rapid Response Survey)**						
School Modality	Closures, remote learning, teacher instructional format	—	RRR W1–W6	RRR W1–W6	—	—
Schedule/Engagement	Hours of schoolwork, stress related to schoolwork, preparedness, reasons for missing school	—	RRR W1–W6	RRR W1–W6	—	—

*Note*. Although the table indicates the full range of waves in which each measure appears, not every item within a measure was administered at every wave listed. In several cases, related items were grouped together for conceptual clarity (e.g., School Climate subscales; KSADS Background Items), but specific items within those groupings may have only been assessed at certain waves. The table reflects measures available in the ABCD 6.0 public release and includes items from the main cohort, the Social Development Substudy, and the COVID Rapid Response Research surveys. Readers should refer to the ABCD Release Notes and item-level documentation for exact wave-by-wave availability.

ABCD = Adolescent Brain Cognitive Development Study; APQ = Alabama Parenting Questionnaire; B = Baseline (ages 9–10); BPM = Brief Problem Monitor (teacher version in K–12); COI 2.0 = Child Opportunity Index, Version 2.0; HOME-SF = Home Observation for Measurement of the Environment Inventory, Short Form; IEP = Individualized Education Program; JVQ = Juvenile Victimization Questionnaire; KSADS = Kiddie Schedule for Affective Disorders and Schizophrenia; METs = Metabolic equivalent of task; MCTQ = Munich Chronotype Questionnaire; METs = Metabolic Equivalent of Task (physical activity metric); MNBS = Multidimensional Neglectful Behavior Scale; NCES = National Center for Education Statistics; PTA = Parent Teacher Association. RRR = Rapid Response Research COVID-19 surveys; SD Substudy = Social Development Substudy (ABCD ancillary study); SEDA = Stanford Education Data Archive; SRPF = Social Relationships and Positive Feelings Questionnaire; Y1–Y6 = 1-year through 6-year follow-up assessments; W = Wave.

### 1.4 Current review

Because educational constructs are dispersed across multiple components of the ABCD Study, they can be difficult to identify and fully leverage. Therefore, the current review synthesized education-related ABCD findings to identify overarching patterns in how school environments relate to adolescent behavior and neurodevelopment, and in turn, how these processes predict academic outcomes. Rather than evaluate the methodological rigor of each ABCD publication, the goal of this review was to characterize the major themes emerging across the literature. By surveying studies that varied in design, analytic approach, and topic area, this review also highlights the many ways in which ABCD can be leveraged to examine the interplay among school experiences, contextual factors, and adolescent cognitive, socio-emotional, and health development. Clarifying which school and neighborhood educational indicators function as risks and which as protective or promotive, and for whom, will be essential for developing targeted interventions that buffer adversity and support resilience. These aims are grounded in broader conceptual perspectives introduced earlier (i.e., educational neuroscience and bioecological theory), which emphasize the importance of examining educational environments within multilevel developmental frameworks, an approach that is well supported by the ABCD Study.

## 2 Method

We conducted a narrative review of ABCD Study peer-reviewed publications using the official publication repository maintained on the ABCD Study website (https://abcdstudy.org/research-publications/), which indexes articles citing the ABCD Study from MEDLINE, Web of Science, Scopus, Directory of Open Access Journals, and the National Institutes of Health Library. The search included ABCD Study peer-reviewed manuscripts available as of September 7, 2025. To maximize coverage of school- and education-related constructs, broad and inclusive search terms were used to capture a wide range of studies potentially relevant to educational environments, even if not all were ultimately included. The following keywords were entered into the repository search engine: academic; attending new school; Brief Problem Monitor; changing school; Child Opportunity Index; COVID; detention; discrimination; education; educational opportunity; extracurricular activities; friends; grades; hybrid; leisure reading; NIH Toolbox; oral reading; pandemic; peer behavior profile; peer bullying; peer delinquency; peer network health; peer prosocial; reading for pleasure; remote; resistance to peer influence; school; school attendance; school climate; school disengagement; school discipline; school engagement; school involvement; school lockdown; school safety; Stanford Education Data (SEDA) Archive; Stanford Mental Arithmetic Response Time Evaluation (SMARTE); suspension; teacher; truancy; unfair treatment; picture vocabulary.

Four reviewers (one research assistant, two graduate students, and one ABCD co-investigator) each screened a subset of publications for relevance. Articles were included if they (a) used ABCD Study data and (b) examined educational, school, or academic-related constructs as predictors, correlates, or outcomes (e.g., linked academic data, reading skills, academic grades, perceived school environment; see Table 1). We excluded studies focused solely on non-school or unspecified contexts (e.g., number of close friends, peer behavior outside of school, general discrimination rather than teacher- and other student-specific experiences, general cognition versus crystallized cognition focused on reading and vocabulary skills), studies using caregiver education as the sole educational variable, as well as studies focused on genetic propensity to academic outcomes. Given the broad scope and rapid growth of ABCD publications, screening was designed to prioritize comprehensive coverage rather than inter-rater reliability metrics.

**Fig. 1:**
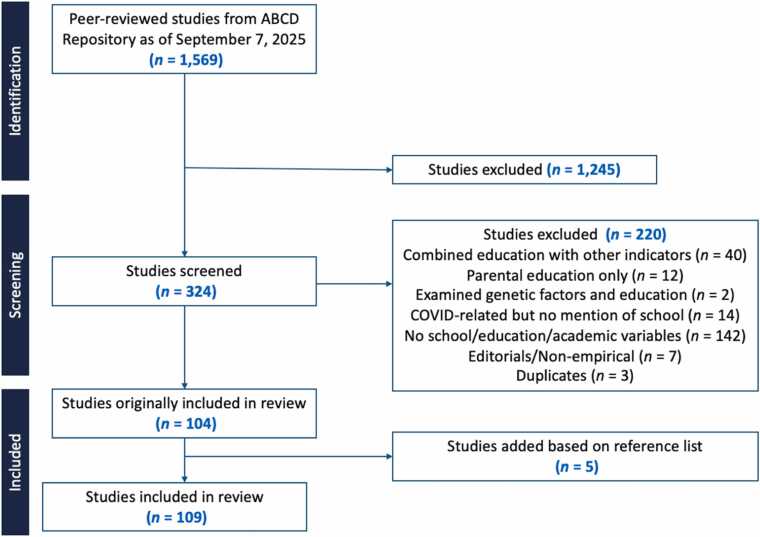
PRISMA Flow Diagram. **Note.** PRISMA = Preferred Reporting Items for Systematic Reviews and Meta-Analyses. Counts reflect peer-reviewed publications identified through the ABCD publication repository as of September 7, 2025.

A PRISMA-style flow diagram summarizing the screening process is presented in Fig. 1. The initial search of the ABCD Study publication repository identified 1569 peer-reviewed manuscripts. After removing studies that did not meet the inclusion criteria, 324 studies remained for full screening. Of those, 220 were excluded according to the predefined exclusion criteria mentioned previously, resulting in 104 studies. An additional 5 studies were identified through reference list searching and prior knowledge of co-authors, yielding a final sample of 109 studies included in the narrative review.

## 3 Results

Across 109 studies (representing slightly less than 7% of all ABCD publications at the time of writing), educational variables functioned as predictors, outcomes, and contextual modifiers. Notably, this included 56 longitudinal and 53 cross-sectional studies, allowing for both cross-sectional associations and emerging evidence of bidirectional processes over time. Table 2 summarizes the main takeaways across studies and indicates the distribution of cross-sectional and longitudinal designs within each section. Longitudinal studies are denoted with an asterisk (*) throughout the text. As illustrated in the graphical abstract, ABCD findings indicate that supportive and adverse educational environments operate as multilevel systems that both shape and respond to neurodevelopmental and behavioral processes.

**Table 2:** Summary of Key Conceptual Takeaways from Education-Related ABCD Study Findings.

**Section**	**Prior Knowledge**	**Key Conceptual Takeaway**	**Studies**
**3.1 Supportive School Environments and Adolescent Development**			
**3.1.1**	Family socioeconomic status is a primary driver of neurodevelopment	Educational environments contribute unique variation to brain development and are not simply proxies for socioeconomic status	8 studies: 4 cross-sectional; 4 longitudinal
**3.1.2**	Associations between neurodevelopment and behavior are not uniform across contexts	Educational factors also help explain why associations between brain networks and cognitive performance differ across socioeconomic contexts	3 studies: 3 cross-sectional; no longitudinal
**3.1.3**	Brain development supports learning and cognition	Reading and brain development influence one another over time, with early biological factors shaping these relationships	18 studies: 14 cross-sectional; 4 longitudinal
**3.1.4**	Supportive environments protect against mental health problems	Supportive educational contexts function across multiple levels to buffer the emergence of mental health symptoms and shape responses to adversity	20 studies: 8 cross-sectional; 12 longitudinal
**3.2 Adverse School Experiences and Developmental Risk Pathways**			
**3.2.1**	Child adversity increases risk for internalizing and externalizing problems	Adverse school experiences increase risk for mental health concerns through cumulative, bidirectional processes	19 studies: 6 cross-sectional; 13 longitudinal
**3.2.2**	Child adversity increases substance use risk	Adverse school experiences appear to accelerate early substance use initiation rather than escalation	4 studies: 2 cross-sectional; 2 longitudinal
**3.2.3**	Child adversity impacts physical and behavioral health	Adverse school experiences are also linked to other health risks, which, in turn, are associated with poorer academic functioning	4 studies: 4 cross-sectional; 0 longitudinal
**3.2.4**	School disruptions affect learning and well-being	Remote learning was linked to lower school engagement and adverse mental health outcomes, but these effects were not uniform and were strongly shaped by family conditions	12 studies: 1 cross-sectional; 11 longitudinal
**3.3 Behavioral Health and Subsequent Educational Outcomes**			
**3.3.1**	Socioeconomic status is associated with academic performance	Brain development plays an important role in academic performance, with associations varying across socioeconomic contexts	11 studies: 2 cross-sectional; 9 longitudinal
**3.3.2**	Adolescence involves changes in sleep and circadian rhythms	Modifiable school structures, such as start times and daily schedules, shape sleep, and, in turn, academic outcomes	6 studies: 2 cross-sectional; 4 longitudinal
**3.3.3**	Technology influences youth behavior	Screen use may reflect broader patterns of disengagement and contextual disadvantage rather than serving as a primary driver of academic outcomes	4 studies: 1 cross-sectional; 3 longitudinal
**3.4 Methodological and Developmental Considerations**			
**3.4.1**	Caregiver and youth reports are often the gold standard in clinical research	The ABCD Study’s multi-informant and multimethod design provides a unique opportunity to capture different dimensions of adolescents’ experiences and functioning	10 studies: 7 cross-sectional; 3 longitudinal
**3.4.2**	Day-to-day routines can be very different during the academic school year compared to the summer	The timing of assessment relative to the school context may influence observed neurodevelopmental patterns	1 cross-sectional study; 0 longitudinal

Findings are organized into four broad domains. First, supportive school environments were associated with multiple aspects of adolescent development, including neurodevelopment, reading, and emotional and behavioral functioning (Section 3.1). Second, adverse school experiences functioned as meaningful sources of stress that contributed to developmental risk, particularly with co-occurring stress exposures (Section 3.2). Third, neurodevelopmental, behavioral, and health processes were associated with academic outcomes, highlighting the bidirectional links between education and adolescent development (Section 3.3). Finally, we discuss methodological and developmental considerations, including multi-informant measurement and assessment timing, highlighting the breadth of constructs available for educational neuroscience research (Section 3.4). Additional study details (constructs, design, covariates) are summarized in Table S1.

### 3.1 Supportive school environments and adolescent development

Supportive educational environments have long been recognized as important for youth development. ABCD Study findings further show that (1) supportive school environments are not simply proxies for socioeconomic status but are independently associated with brain development, (2) school-brain associations vary across socioeconomic contexts, (3) reading and brain development influence one another over time, and (4) schools appear to buffer the effects of prior child adversity on mental health outcomes.

#### 3.1.1 Educational environments contribute unique variation to brain development and are not simply proxies for socioeconomic status (SES)

Family socioeconomic conditions have long been central to models of neurodevelopment, with extensive evidence linking socioeconomic disadvantage to differences in brain structure and function (for reviews, see Baysarowich et al., 2025; Rakesh, Whittle et al., 2023). Building on this work, ABCD research demonstrates that educational environments contribute additional, non-overlapping variation in brain development rather than serving as simple proxies for socioeconomic conditions. Across studies, more positive school climates, including greater teacher support and student involvement in school activities, were associated with greater cortical myelination, higher within-cingulo-opercular network connectivity, decreased connectivity between the cingulo-opercular and default-mode networks, as well as greater deviations from group-normative patterns within the posterior default-mode, visual, and control networks (i.e., individual-level differences without a consistent positive or negative direction) (Hong et al., 2021; *Petrican et al., 2024; Rakesh, Zalesky et al., 2023). Importantly, these findings were over and above the effects of other individual, family, and neighborhood characteristics. Similarly, home, school, neighborhood, and cultural environments each explained unique variance in overall cognitive ability beyond SES (Marotta et al., 2025, Meredith et al., 2022), highlighting that these correlated contexts reflect distinct dimensions of youth experience.

Studies leveraging district- and neighborhood-level educational measures also illustrate how school environments operate independently of SES (*Roy et al., 2024; *Zhou et al., 2025). In longitudinal analyses linking residential history information to geocoded data, living in areas with greater educational resources (e.g., access to high quality early education, advanced placement coursework enrollment, adult educational attainment) was associated with higher cognitive performance, including language, memory, and inhibitory control, as well as larger whole-brain gray matter volume and total cortical surface area, even after adjusting for family income and other neighborhood economic characteristics (*Zhou et al., 2025). Similarly, linking ABCD data to the Stanford Education Data Archive (SEDA), *Roy et al. (2024) found that higher district-level academic achievement was associated with accelerated patterns of white matter development (indexed by brain-age gap, which compares an individual’s chronological age with a machine learning prediction of age based on neuroimaging features), independent of income-to-needs and parental education.

Notably, longitudinal analyses (*Bates et al., 2025) found that while positive school experiences and cortical thickness positively covaried over time, school experiences did not predict subsequent changes in whole-brain cortical thickness, suggesting coordinated development rather than a unidirectional effect. Overall, these findings suggest that educational contexts and neurodevelopment are associated above and beyond SES; however, they do not necessarily indicate causation.

#### 3.1.2 Educational factors also help explain why associations between brain networks and cognitive performance differ across socioeconomic contexts

Importantly, the effects of neurodevelopment are not uniform across contexts. ABCD findings indicate that school characteristics can help explain why associations between brain networks and cognitive performance differ across socioeconomic contexts (Ellwood-Lowe et al., 2021, Hackman et al., 2022, Rakesh et al., 2021). For example, positive school environments attenuated the associations between higher neighborhood disadvantage and lower resting-state connectivity within the cingulo-opercular and frontoparietal networks (Rakesh et al., 2021). These findings suggest that school contexts may buffer the relationship between environmental adversity and brain network organization, which, in turn, can have downstream effects, such as reduced cognitive ability and more mental health concerns.

School characteristics can also help explain why associations between brain networks and cognitive performance differ across socioeconomic contexts. Consistent with prior work, weaker lateral frontoparietal–default mode network (LFPN–DMN) coupling was associated with better nonverbal cognitive performance among youth living above poverty (Ellwood-Lowe et al., 2021). In contrast, *stronger* LFPN–DMN coupling was associated with better performance among youth living in poverty, and this effect depended on attending a public school rather than a charter or private school (Ellwood-Lowe et al., 2021). Although more longitudinal work is needed to fully interpret these findings, differences in resources across school settings likely contribute to how brain-behavior associations are expressed across socioeconomic contexts.

Not all findings, however, support a buffering role of positive school environments in SES-brain associations. Building on prior work linking socioeconomic disadvantage to reduced cortical surface area, Hackman and colleagues (2022) originally hypothesized that positive school climates would attenuate this association. Instead, socioeconomic differences in whole-brain cortical surface area varied by school climate: more positive school climates were associated with smaller cortical surface area among lower-income youth, but not among higher-income youth. The authors suggest that this pattern may reflect differences in the timing of neurodevelopment across SES (e.g., earlier cortical thinning or accelerated development), though these findings cannot be fully interpreted without longitudinal data. Together, these findings highlight that the role of school environments in neurodevelopment may not be uniformly protective but instead differ in complex ways depending on broader socioeconomic context.

#### 3.1.3 Reading and brain development influence one another over time, with early biological factors shaping these relationships

Neurodevelopment has increasingly been linked to reading skills (for reviews, see Chyl et al., 2021; Pınar et al., 2025). ABCD research supports bidirectional associations, with reading and brain development influencing one another over time, with early biological factors shaping these relationships. Across numerous cross-sectional analyses, differential patterns of cortical structure, white matter organization, and functional connectivity were associated with reading and vocabulary skills (Byington et al., 2023, Carrión-Castillo et al., 2023, Coccaro et al., 2024, Eckert et al., 2023; Fekson et al., 2023; Langensee et al., 2023; Owens et al., 2020; Palmer et al., 2021; Rauschecker et al., 2025; Roy, Richie-Halford et al., 2024; Tomasi and Volkow, 2021; Wang et al., 2021). More specifically, studies found positive associations between reading performance and greater whole-brain grey matter volume as well as greater cortical area in the lateral temporal, inferior parietal, and inferior frontal lobes (Langensee et al., 2023, Rauschecker et al., 2025). Together, these findings identify potential neural systems associated with reading, but do not establish the direction of these relationships.

Emerging longitudinal evidence provides some initial support for bidirectional associations between reading and brain development. Specifically, reading time was positively associated with larger brain volumes in 22 areas two years later, including the bilateral prefrontal, temporal, insula regions, and left caudate (*Li, Zhao et al., 2024). In turn, several brain regions, including the circular sulcus of the insula, the right middle temporal gyrus, the right inferior temporal sulcus, the left putamen, and the right thalamus were positively associated with subsequent time spent reading two years later (*Li, Zhao et al., 2024). Mediation analyses further indicated that total brain volume and intracranial volume mediated associations between earlier reading for pleasure and crystallized cognition (i.e., vocabulary and reading performance; *Sun et al., 2024). These findings provide early evidence that reading and neurodevelopment may influence one another over time, although additional longitudinal work is needed to confirm these bidirectional pathways.

Early biological and developmental factors appear to further shape these brain-reading relationships, controlling for socioeconomic conditions. Lower gestational age was associated with smaller cortical and subcortical volumes in the fronto-parieto-temporal areas, fusiform gyrus, cingulate cortex, insula, postcentral gyrus and the right hippocampus, thalamus, and pallidum, which, in turn, were associated with weaker reading and language abilities (*Ma et al., 2022). Among those born preterm or early-term, early adolescents with below average reading and verbal skills were more likely than those with average skills to have smaller total cortical volumes and surface areas (*Menu et al., 2025). Maternal prenatal hypertensive disorders were also associated with weaker language outcomes via lower birth weight and higher body mass index (BMI) rather than through cortical alterations (Ahmed et al., 2024). Finally, later attainment of motor milestones was also associated with lower reading and vocabulary skills at ages 9–10 (Zhuo et al., 2022). Collectively, these findings indicate that brain development and reading development likely influence one another over time, with early biological factors shaping these developmental trajectories.

#### 3.1.4 Supportive educational contexts function across multiple levels to buffer the emergence of mental health symptoms and shape responses to adversity

Supportive environments have long been recognized as beneficial for adolescent mental health (for reviews, see Aldridge and McChesney, 2018; Marth et al., 2022). ABCD research further shows that positive educational contexts, including school experiences as well as neighborhood- and school-level educational conditions, can buffer the emergence of mental health symptoms. ABCD studies using both cross-sectional and longitudinal designs showed that supportive school environments, including positive climate, engagement, and connectedness, reduced the likelihood of internalizing, externalizing, and inattention symptoms, callous-unemotional traits, suicidal ideation, psychotic-like experiences, and improved emotional regulation (*Brieant and Simmons, 2025; Crumly-Goodwin and Samek, 2024; Hackman et al., 2022; *Keyin et al., 2025; *Logan and Lewis-de los Angeles, 2025; *Park et al., 2024; Qiu and Liu, 2023; Thapaliya et al., 2021, Thapaliya et al., 2024; Vargas et al., 2025; *Smith and Stamoulis, 2023; *Sun et al., 2024; *Wallace and Conner, 2025; *Wen et al., 2023; *Yang, Tuy et al., 2025). These effects were generally small (i.e., typically explaining a small proportion of variance) but consistent across reporters and domains.

Beyond these direct associations, more nuanced patterns emerged: supportive school contexts modestly attenuated associations between prior adverse childhood experiences and later internalizing and externalizing problems, controlling for individual and family characteristics (*Logan and Lewis-de los Angeles, 2025; *Martínez, Damme et al., 2024; *Petrican et al., 2024). For example, *Logan and Lewis-de los Angeles (2025) found that having caring teachers and liking school buffered the negative impact of prior adverse experiences on a range of symptoms, including anxiety, depression, rule-breaking behavior, aggression, social problems, and somatic complaints.

In addition to these proximal school experiences, broader educational characteristics also contributed to youth mental health. Educational resources that improve mental health go beyond self-reported school climate, including neighborhood conditions, school-based ethnic density, and neighborhood educational opportunities (Harris et al., 2025, Urbina-Johanson et al., 2025, Vargas et al., 2025). For example, a higher proportion of same-race and ethnicity peers at school was associated with lower levels of mental health problems among Black youth and Latino/a/e youth from immigrant families, highlighting the potential protective role of school ethnic density for some groups (Urbina-Johanson et al., 2025). Prior work also found that higher school-level academic performance predicted better decision-making skills four years later (*Gonzalez et al., 2025), and higher neighborhood educational opportunity (e.g., access to quality daycares, adult educational attainment) corresponded with slightly lower concurrent reward-seeking behaviors (Harris et al., 2025). Accordingly, supportive educational environments are likely best understood as multi-level contexts that shape risk for mental health outcomes in numerous ways.

### 3.2 Adverse school experiences and developmental risk pathways

In contrast to supportive school environments, which can promote positive development, adverse school experiences may also function as sources of stress. These school-based experiences include events such as lockdowns due to potential threats, unfair treatment by teachers and other students, and disciplinary actions (see Table 1 for a comprehensive list).

The current section summarizes how adverse school experiences relate to (1) emotional and behavioral concerns through cumulative stress processes, (2) early substance use initiation, (3) health indicators and behavioral coping pathways, and (4) disruptions to school experiences during the early stages of the COVID-19 pandemic. Together, these subsections illustrate how adverse school experiences contribute to developmental risk through interconnected emotional, behavioral, and health pathways.

#### 3.2.1 Adverse school experiences increase risk for mental health concerns through cumulative, bidirectional processes

Exposure to stressful life events is a well-established risk factor for internalizing and externalizing problems (for reviews, see March‑Llanes et al., 2017; Wade et al., 2022). ABCD research extends this work by showing that negative school experiences can function as meaningful sources of stress that rarely occur in isolation. Across studies, school-based stressors were consistently associated with higher levels of internalizing and externalizing concerns (*Brislin et al., 2025; *Conley et al., 2024; *Conley, Hernandez et al., 2023; Conley, Rapuano et al., 2023; *Kong et al., 2025; Menken et al., 2022; *Yang, Tuy et al., 2025). For example, experiencing a school lockdown was associated with increases in anxiety and stress over time, particularly among youth with pre-existing vulnerabilities such as elevated baseline stress or attention-deficit/hyperactivity disorder (*Hullenaar et al., 2025). Similarly, perceived unfair treatment within school settings was linked to elevated internalizing and externalizing symptoms across reporters (*Yan et al., 2025), while peer victimization and school disengagement partially accounted for associations between prior childhood adversity and mental health outcomes (*Feinstein et al., 2024; Loso et al., 2023; Schiff and Lee, 2023).

ABCD findings further indicated that some adverse school experiences are embedded within feedback loops linking structural conditions, behavior, and school responses over time. For example, externalizing behaviors have long been linked to increased risk of school discipline, and longitudinal ABCD evidence suggests that school discipline, in turn, contributes to escalating behavioral problems over time, creating a reinforcing cycle, particularly among Black youth (*Brislin, Choi et al., 2024). Furthermore, exposure to school discipline and perceived unfair treatment in schools were also systematically linked to race, neighborhood opportunity, and other structural conditions, which was not explained by individual behavior (Fadus et al., 2021, Nagata et al., 2021; *Thompson et al., 2025).

Consistent with this broader pattern, decreased school involvement and increased disengagement mediated associations between adverse childhood experiences and increases in internalizing and externalizing problems over time (*Niu et al., 2025), highlighting school disengagement as a potential mechanism linking childhood adversity to worsening mental health. Extending this work, results suggested that externalizing behavior is driven largely by shared environment rather than genetic influences among youth living in lower educational opportunity neighborhoods (*Dash et al., 2023). Accordingly, Dash and colleagues suggest that community-based prevention efforts targeting environmental factors (e.g., school climate, quality, resources, and educational opportunity) may help mitigate externalizing behaviors, rather than focusing solely on individual-level interventions.

Notably, mental health concerns were more commonly observed among youth exposed to stress across multiple settings, including school. While some youth experienced adversity confined to family or neighborhood contexts, school-based adversity rarely occurred on its own and was typically observed alongside stress in multiple settings (*Conley et al., 2024; *Conley, Hernandez et al., 2023; Conley, Rapuano et al., 2023; *Martinez, Cai, et al., 2024). This suggests that school adversity contributes to the accumulation of stressors rather than operating in isolation. This pattern is also consistent with stress sensitization, suggesting that school stressors may compound risk among youth with prior adversity.

Overall, these findings demonstrate that adverse school experiences may be unequally distributed and may both reflect and reinforce behavioral risk. Findings also suggest that school-based stressors do not operate in isolation but instead contribute to stress accumulation that increases risk for maladjustment. The accumulation and co-occurrence of stressors within and outside the school setting may be particularly consequential for adolescent emotional and behavioral outcomes.

#### 3.2.2 Adverse school experiences appear to accelerate early substance use initiation rather than escalation

It is well established that a variety of individual, social, and environmental factors influence adolescent substance use, including peer use, family environment, school context, and broader socioeconomic conditions (for a review, see Trucco, 2020). ABCD research highlights specific mechanisms through which school experiences shape early initiation. Across two ABCD studies, school context appears to be more strongly related to the *timing* of substance use initiation rather than to progression once substance use has begun. More specifically, unsupportive school environments were associated with more favorable beliefs about alcohol prior to initiation and with earlier experimentation over time (*Choi et al., 2025; Sanchez et al., 2023); however, perceived school environment did not predict escalation to more advanced use (*Choi et al., 2025). These findings suggest that school environments may act as a key entry point for substance use rather than influence escalation once use has begun.

In addition to perceived school climate, other adverse school experiences showed strong associations with early substance use initiation. Youth with a history of school discipline were more likely to initiate substance use by early adolescence, with the effects of school discipline exceeding the strength of most other contextual factors (*Green et al., 2024). Similarly, perceived unfair treatment by teachers was associated with substantially higher odds of subsequent substance use, based on both self-report and hair toxicology, even after adjusting for mental health and sociodemographic factors (Jelsma et al., 2025). These findings suggest that universal school-based interventions targeting structural features of the school environment may be particularly important for preventing early initiation of substance use, rather than altering trajectories once use has begun. However, further longitudinal work will be needed as ABCD participants age into later adolescence and emerging adulthood, particularly given that current substance use rates within the ABCD Study are low and may limit detection of escalation.

#### 3.2.3 Adverse school experiences are also linked to other health risks, which, in turn, are associated with poorer academic functioning

Familial and environmental stress exposures are robustly linked to numerous health outcomes (for reviews, see Bush et al., 2026; Hughes et al., 2017). ABCD findings further suggest that adverse school experiences are also associated with health problems within adolescence (Allen et al., 2022, Li et al., 2024, Raney et al., 2023, Waltzman et al., 2024). For example, perceived unfair treatment by other students was associated with greater risk for binge-eating behaviors and meeting criteria for binge-eating disorder (Raney et al., 2023), suggesting that school-based stress may influence maladaptive coping behaviors.

Additional research indicated that educational and health factors are closely intertwined. Lower neighborhood educational resources were among the strongest predictors of youth BMI (Allen et al., 2022), while higher BMI was, in turn, associated with poorer academic functioning, including lower reading skills and grades (Li, Thomas et al., 2024). Additionally, youth with a history of head injury or traumatic brain injury showed increased risk for poorer academic performance, disciplinary involvement, and receipt of special education services (Waltzman et al., 2024), underscoring the interconnected nature of health and school experiences. However, this evidence is based on cross-sectional analyses, and additional longitudinal research will be needed to clarify the (bi)directionality of these associations over time. Overall, these findings highlight school-based adversity as an important and underrecognized context for understanding links between health and academic functioning.

#### 3.2.4 Remote learning was linked to lower school engagement and adverse mental health outcomes, but these effects were not uniform and were strongly shaped by family conditions

COVID-19 disruptions affected many domains of adolescent life, and ABCD offers a distinct opportunity to examine how abrupt changes in school structure intersected with family, neighborhood, and other stressors. Another article in this special issue (See (Gonzalez et al., under submission)) synthesizes the broader ABCD COVID-19 literature; here, we briefly highlight education-related disruptions, primarily drawing on the longitudinal rapid response assessments from May 2020 to March 2021.

During the first year of the pandemic, remote learning was widespread and associated with lower enjoyment of school as well as reductions in time spent on core subjects (*Guillaume et al., 2022). At the same time, family context played a key role in shaping these experiences: greater parental involvement was linked to more positive school attitudes, whereas pre-pandemic family hardship and conflict predicted greater difficulty completing homework (*Guillaume et al., 2022; *Gonzalez et al., 2023). Changes in academic experiences also intersected with mental health: worry about schoolwork was associated with early pandemic depressive symptoms (*Kiss et al., 2022), while returning to in-person instruction was associated with better mental health among youth with higher pre-pandemic ACEs (*Raney et al., 2024). Youth with ADHD additionally experienced more remote-learning difficulties than matched peers (*Rosenthal et al., 2022). These findings indicate that remote learning altered core features of schooling (e.g., engagement, structure, support), with downstream implications for development.

Although many ABCD COVID-19 studies focused on domains outside of schooling, such as sleep, screen time, physical activity, overall distress, and substance use (*Hamatani et al., 2022; Hunt et al., 2024; *Kiss et al., 2023; *Pelham et al., 2021; *Stinson et al., 2021; *Yip et al., 2022), these behavioral shifts may partially reflect changes in school structure, daily routines, and academic expectations. Notably however, one study found that adolescent internalizing concerns were more strongly linked to COVID-related financial strain rather than school closures specifically (*Xiao et al., 2023), underscoring the importance of contextual stressors that may modify the impact of pandemic-related educational disruptions on adolescent development.

### 3.3 Behavioral health and subsequent educational outcomes

Academic functioning is an important marker of adolescent development and has long been linked to cognitive, behavioral, health-related, and financial outcomes. ABCD findings build on this work by showing that academic functioning is closely linked to neurodevelopment and everyday experiences across adolescence. This section focuses on three primary areas: (1) brain development and academic outcomes, (2) school schedules, sleep, and academic performance, and (3) technology use and subsequent school disengagement. Notably, comprehensive educational attainment outcomes (e.g., graduation rates) are not yet available in ABCD.

#### 3.3.1 Brain development plays an important role in academic performance, with associations varying across socioeconomic contexts

Socioeconomic conditions have long been associated with educational outcomes (for a review, see Zimmerman et al., 2015). ABCD findings indicate that neurodevelopmental processes are closely linked to academic performance, even after accounting for SES (*Li et al., 2025; *Rakesh et al., 2025; *Yang et al., 2023; *Yang, Zhou et al., 2025). For example, longitudinal work showed that stronger intrinsic connectivity between the frontoparietal network and the striatum as well as increased functional connectivity between the cingulo-opercular network and right hippocampus were associated with better academic performance across time, controlling for indicators of SES (*Yang et al., 2023; *Yang, Zhou et al., 2025). Other work demonstrated that larger whole-brain cortical surface area and subcortical volume statistically mediated the association between greater greenspace exposure and better academic performance, even after accounting for income-to-needs (*Li et al., 2025). Conversely, greater increases in within-sensorimotor network connectivity mediated the association between lower parental educational attainment and lower school grades across three years (*Rakesh et al., 2025). These findings suggest that interventions targeting neurodevelopment may have downstream effects on academic performance.

Importantly, these associations also varied across family and socioeconomic contexts (Alnæs et al., 2020; *Devakonda et al., 2024; Ellwood-Lowe et al., *2022; *Elton et al., 2025; *Langensee et al., 2024; Tomasi and Volkow, 2021; *Zhou et al., 2025). In cross-sectional work, lower total cortical volume, lateral occipital cortical volume and thickness, and bilateral lingual thickness were simultaneously correlated with economic deprivation, neighborhood violence, and lower academic grades and language performance (Alnæs et al., 2020), likely reflecting a pattern of socio-cognitive stratification and aligning with evidence of earlier or more rapid brain maturation among youth from lower SES backgrounds. In addition, higher LFPN-DMN connectivity predicted better grades among youth living below the poverty line but showed the opposite pattern among youth above the poverty line (*Ellwood-Lowe et al., 2022). *Ellwood-Lowe and colleagues also found that baseline cognitive performance was more strongly correlated with later academic performance among children living above the poverty line compared to those below it. The weaker relationship between cognitive ability and academic success may reflect external stressors that act as barriers to learning, reduced access to educational resources, and lower instructional quality.

Further longitudinal work identified additional trade-offs across developmental domains. For example, youth exposed to prior childhood adversity exhibited reduced cortical-subcortical connectivity, which buffered emotional distress but was linked to poorer academic performance (*Elton et al., 2025). At the same time, family socioeconomic resources, such as income and parental education, remained strong predictors of academic outcomes across development (*Langensee et al., 2024; Tomasi and Volkow, 2021; *Zhou et al., 2025). These findings suggest that associations between neurodevelopment and academic outcomes are not uniform but depend on family and socioeconomic context. Importantly, they also indicate that the same neurodevelopmental patterns may support different outcomes across domains, such as adaptations that buffer emotional risk but potentially come at the cost of academic performance.

#### 3.3.2 Modifiable school structures, such as start times and daily schedules, shape sleep, and, in turn, academic outcomes

Adolescence is characterized by well-established changes in sleep and circadian rhythms, including a developmental shift toward later bedtimes and a natural preference for later wake times (for reviews, see Tarokh et al., 2019; Crowley et al., 2007). However, school schedules often conflict with these biological shifts, requiring adolescents to wake up earlier than their circadian rhythms would naturally support. ABCD research provides further evidence that educational environments shape biological regulation with downstream developmental and academic consequences. For example, at ages 11–12, youth reported approximately 45 min less sleep on school nights than on weekends (*Wang et al., 2025), reflecting a mismatch between school schedules and adolescent sleep patterns. Across studies, school-imposed schedules appeared to contribute to circadian misalignment (i.e., difference in sleep timing between school days and weekends), which was associated with lower reading scores, poorer academic grades, and changes in emotional functioning over time (Li, Thomas et al., 2024; *Wallace, Aguinaldo et al., 2025; *Yang et al., 2023).

Additional evidence from the ABCD Study suggests that sleep timing may be particularly important: later bedtimes were more strongly associated with poorer academic performance than sleep duration across follow-up timepoints (*Zhang et al., 2025). Moreover, later school start times were moderately associated with longer weekday sleep, though primarily among White youth (Yip et al., 2025). Overall, these findings indicate that school structures shape daily physiological rhythms linked to both health and academic outcomes, highlighting the role of educational policy decisions in shaping biological processes and later academic outcomes during adolescence.

#### 3.3.3 Screen use may reflect broader patterns of disengagement and contextual disadvantage rather than serving as a primary driver of academic outcomes

In addition to school- and family-based experiences, societal changes in everyday routines likely also shape educational engagement. Technology use is an increasingly important contextual factor within these environments, although the developmental consequences remain incompletely understood (for a review, see Dibben et al., 2023). Longitudinal evidence from the ABCD Study suggests that technological use likely both reflects and co-occurs with negative school experiences: youth reporting greater unfair treatment by a teacher were more likely to follow a higher screen-use trajectory over time (8% versus 3%; *Shao et al., 2023), and problematic mobile phone use predicted lower school involvement at follow-up (*Xu et al., 2025). Similarly, more weekday and weekend non-academic screen use was concurrently associated with worse academic performance at ages 9–10, although socioeconomic factors remained the strongest predictors of academic outcomes (Paulich et al., 2021).

Importantly, additional longitudinal work using causal modeling indicated that, although screen use is prospectively associated with poorer reading performance, duration of screen use was likely an outcome, not a cause of externalizing symptoms (*Li et al., 2024). Together, these findings indicate that screen use is associated with academic and behavioral outcomes, but the small effect sizes and strong links to socioeconomic and contextual factors suggest that screen use represents a secondary pathway rather than a primary developmental driver of school disengagement and other academic outcomes.

### 3.4 Methodological and developmental considerations

Educational experiences and neurodevelopment are dynamic and can be captured across multiple reporters and measurement approaches. The following subsections highlight key methodological and developmental considerations in ABCD educational research, focusing on (1) multi-informant measurement and (2) the timing of assessments within school contexts.

#### 3.4.1 The ABCD Study’s multi-informant and multimethod design provides a unique opportunity to capture different dimensions of adolescents’ school experiences and functioning

The ABCD Study’s integration of multiple informants and assessment approaches within the same sample provides a unique opportunity to directly compare how different types of measures capture adolescents’ school experiences, academic performance, and related developmental outcomes. School context, in particular, is not equally observable across informants, underscoring the limitations of relying on any single method of assessment.

Consistent with this notion, ABCD Study findings show that results differed depending on who reported the information. For example, youth-caregiver concordance on school victimization was limited (Tang et al., 2025), highlighting the constraints of relying on caregiver reports for experiences that may occur outside their supervision. Teachers also appear to provide a unique perspective: peer support modestly buffered the effects of peer victimization on teacher-reported, but not youth- or caregiver-reported externalizing symptoms (Schiff and Lee, 2023). Incorporating teacher reports was also shown to improve ADHD identification (Cordova et al., 2022; *Weigard et al., 2023), reflecting the importance of school-based observations for understanding attention and behavior in academic settings. Similarly, multitrait-multimethod analyses demonstrated that including teacher reports in the assessment of adolescent psychopathology reduced shared variance across symptoms, thereby weakening support for a general factor of psychopathology (analogous to *g* in intelligence; Watts et al., 2022). Overall, these findings underscore that early adolescents’ functioning cannot be fully captured by any single informant, with teacher reports providing critical insight into behavior in academic contexts.

Beyond informant differences, variation in measurement approaches also shaped conclusions, further reinforcing the need for multiple methods to capture functioning within school contexts. When comparing the utility of caregiver-reported DSM-5 ADHD classifications versus NIH Research Domain Criteria profiles, caregiver-reported DSM-5 ADHD classifications more strongly predicted academic performance, whereas NIH Research Domain Criteria profiles better captured ADHD-related psychosocial functioning (Pintos Lobo et al., 2025).

The integration of self-report, caregiver-report, and behavioral task measures in the ABCD Study also allows for the examination of complex constructs that are difficult to capture with a single method. For example, findings indicated that self-regulation is not well represented by a single general factor, and instead, reflects multiple components with distinct functional relevance (i.e., hot, cool, and executive functioning; *Marek et al., 2025). Moreover, only the cognitive control-related dimensions (cool and executive functioning) predicted better academic performance over time (*Marek et al., 2025), suggesting that top-down components of self-regulation may be particularly relevant for academic performance, whereas bottom-up components may be less predictive. These patterns that may not have been identified without integrating cross-method indicators of self-regulation.

Finally, the ABCD Study enables direct comparisons between subjective and more “objective” measures. For example, differences across self-reported and device-based sleep measures indicated that associations with school-related outcomes may vary depending on measurement approach (Rohr et al., 2025; *Wang et al., 2025), highlighting the importance of future work to determine biases in self-report versus misclassification by accelerometry. Associations between language development and reporting discrepancies further illustrate how methodology can shape conclusions about school-related behaviors and outcomes. For example, higher picture vocabulary scores were associated with lower odds of underreporting social media use (Zhao et al., 2025), suggesting that language ability may influence the accuracy of self-reported behaviors, which are often examined in relation to school functioning.

#### 3.4.2 The timing of assessment relative to the school context may influence observed neurodevelopmental patterns

Adolescents’ daily routines vary across the school year. Emerging ABCD Study evidence suggests that the timing of study visits may influence observed neurodevelopmental patterns, highlighting a novel yet underexplored source of measurement variability. More specifically, Hu et al., (2023) found that fMRI scan timing relative to school schedules (i.e., school days versus weekends, during the school year versus summer vacation, and at different times of day) was associated with differences in functional connectivity. For example, participants scanned during the school year exhibited lower global network organization (i.e., clustering, robustness, and stability) and reduced connectivity across the somatomotor, attention, and temporoparietal networks, compared to those scanned during school vacations. The authors hypothesized that these differences may partly reflect shorter sleep duration during the school year.

Although preliminary, these findings point to a potentially important and underexamined issue: school-related factors, such as time of year or proximity to academic demands, may introduce variability in neuroimaging outcomes. Future work should examine whether associations between educational experiences and other developmental outcomes are robust to variation in assessment timing, and whether accounting for timing helps explain observed variability.

## 4 Summary of ABCD findings

Although children and adolescents spend most of their waking hours in school, fewer than 7% of ABCD Study publications have examined education variables as predictors, outcomes, and contextual modifiers. Despite this limited focus, existing ABCD research demonstrates that school experiences are associated with adolescent development across multiple neurodevelopmental, emotional, and health domains, often through shared pathways involving stress, behavior, and daily routines. Overall, findings converged on a consistent pattern: educational contexts function as both protective and risk factors and are involved in reciprocal processes linking school environments and adolescent outcomes.

Supportive and adverse school experiences were both associated with adolescent development, but in distinct ways. Supportive school environments (e.g., greater involvement, supportive teachers) were associated with modestly better mental health, more favorable neurodevelopmental patterns, and healthier daily behaviors. In contrast, adverse school experiences, including exclusionary discipline, perceived unfair treatment, and disengagement, were associated with elevated mental health concerns, earlier substance use initiation, and risk for poorer health outcomes. Importantly, these associations did not operate in isolation, as negative school experiences often co-occurred with adversity in other settings, with poorer outcomes observed among youth exposed to multiple stressors.

Findings also point to shared pathways underlying these associations. For example, daily routines shaped by school timing were linked to both academic and health outcomes, and stress-related processes cut across domains, while school-based adversity was also associated with mental health concerns, behavioral risk, and health-related behaviors. In some cases, these relationships were reciprocal, suggesting that youth behavior and school experiences may reinforce one another over time, placing adolescents on either positive or negative developmental trajectories.

Importantly, the ABCD Study has advanced educational neuroscience research by integrating educational experiences with multi-modal measures of brain development, behavior, and health within a large, diverse, longitudinal sample. This approach allows for the examination of educational contexts as meaningful developmental environments, rather than proxies for socioeconomic status, and supports the identification of small but consistent associations across domains. ABCD findings further demonstrate that neurodevelopmental processes are closely linked to academic functioning, with brain-based differences associated with variation in learning and performance over time. Importantly, these associations are not uniform but vary across socioeconomic and contextual conditions.

Several features of the ABCD Study also strengthen the interpretation of these findings. The large sample size affords substantial statistical power, and the longitudinal design enables the examination of developmental processes over time. The study’s scale, repeated assessments, and linkage to external data sources further enable the investigation of multi-level influences spanning individual, school, and neighborhood contexts. In addition, analytic approaches such as covariate adjustment, sensitivity analyses, and within-family comparisons offer opportunities to strengthen inference within an observational framework. Together, these features support the identification of consistent patterns that can inform future experimental and prevention-focused research.

### 4.1 Limitations of the current review

Several limitations of this review warrant discussion. A primary limitation is the deliberate decision to prioritize breadth over depth, so that this review can also serve as a resource for researchers seeking to incorporate educational variables into future ABCD analyses. As a result, the breadth of constructs included in the current review limited the extent to which detailed, study-specific results could be provided for every finding. Instead, we aimed to highlight key takeaways and synthesize consistent patterns across studies, and our results prioritize integration over exhaustive detail of the specific findings.

Relatedly, substantial heterogeneity in analytic approaches and reported metrics limited our ability to systematically extract or harmonize effect sizes, which limits direct comparison of magnitude across results. Future work using formal meta-analytic approaches will be important for quantifying effect size magnitude more precisely. Educational constructs were also operationalized differently across studies (e.g., school climate, engagement, and contextual indicators), which may further limit comparability across the literature. At the same time, our approach provides a foundation for future work by clarifying how educational variables have been operationalized and identifying opportunities to expand their use, particularly within educational neuroscience research. More focused reviews on fewer topics may also provide greater depth within specific domains or outcomes.

In addition, as the ABCD Study is observational, findings summarized here are subject to limitations in causal inference. At the same time, the breadth of measures available in the ABCD Study allows for adjustment for a wide range of individual, family, and contextual factors, strengthening confidence in observed associations relative to many prior studies. Finally, as ABCD is an ongoing study, the literature is rapidly evolving, and findings summarized here may be extended or refined as additional waves of data become available.

## 5 Future directions

As ABCD Study participants transition into later adolescence and early adulthood, the study will offer expanded opportunities to examine how educational pathways unfold alongside neurodevelopment, mental health, and social functioning. At the same time, several features of the current ABCD dataset (e.g., linked external data, longitudinal school information, and emerging constructs) remain underutilized and provide important opportunities to advance the study of educational environments as multilevel developmental systems.

The sections that follow highlight (1) opportunities to better capture educational context and opportunity at scale using linked datasets and longitudinal school information, and (2) emerging constructs that will support the study of increasingly diverse educational and workforce pathways.

### 5.1 Capturing educational opportunity at scale

A key opportunity for future ABCD research involves leveraging already linked educational datasets and longitudinal school information to better capture how educational contexts shape development over time. ABCD includes rich self-, caregiver-, and teacher-report measures, and researchers can incorporate external indicators of educational opportunity that situate youth within broader school and district environments. Notably, the ABCD Study collects detailed school information from participants and their caregivers at every assessment timepoint. This information is matched to the National Center for Education Statistics (NCES) database (https://nces.ed.gov/ccd/schoolsearch/), which assigns unique school and district identifiers to every federally funded U.S. school. Because NCES IDs are widely used across educational datasets, they provide a powerful tool for connecting ABCD participant information to rich contextual information about their educational environments.

Building on this infrastructure, a small number of ABCD studies (*n* = 3) have begun to integrate data from the Stanford Education Data Archive (SEDA; https://edopportunity.org/). SEDA compiles standardized test scores in reading/language arts and mathematics, along with demographic and contextual variables, for nearly every public school and district in the United States. By harmonizing state-specific achievement data onto a common national scale, SEDA enables direct comparisons of academic performance and learning rates across educational contexts (Fahle et al., 2021). Although these initial studies demonstrate the value of incorporating linked educational data within the ABCD Study, such approaches remain underutilized. By integrating SEDA with individual-level ABCD Study data, future research can situate participants within broader educational and neighborhood contexts and examine how these contexts relate to neurodevelopmental, behavioral, emotional, and health outcomes over time. Additional linked datasets are described in Cardenas-Iniguez et al. (2024) and ABCD Study documentation, including neighborhood-level educational resources.

**Fig. 2:**
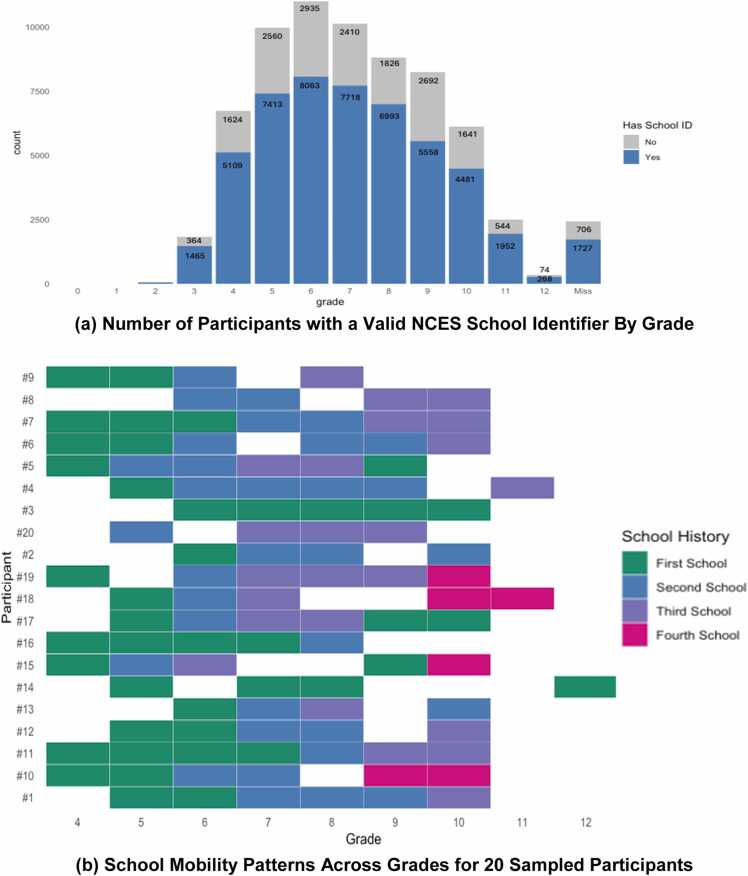
Coverage of Linked School Identifiers and Examples of School Trajectories. **(a) Number of Participants with a Valid NCES School Identifier By Grade. (b) School Mobility Patterns Across Grades for 20 Sampled Participants.***Note.* Panel (a) displays the number of ABCD participants with a valid National Center for Education Statistics (NCES) school identifier at each grade level, highlighting substantial variation in coverage across grades. Panel (b) illustrates school mobility patterns for a randomly selected subset of 20 participants, showing how individual students move across one or more schools over time. Miss = Indicates missing grade information.

**Fig. 3:**
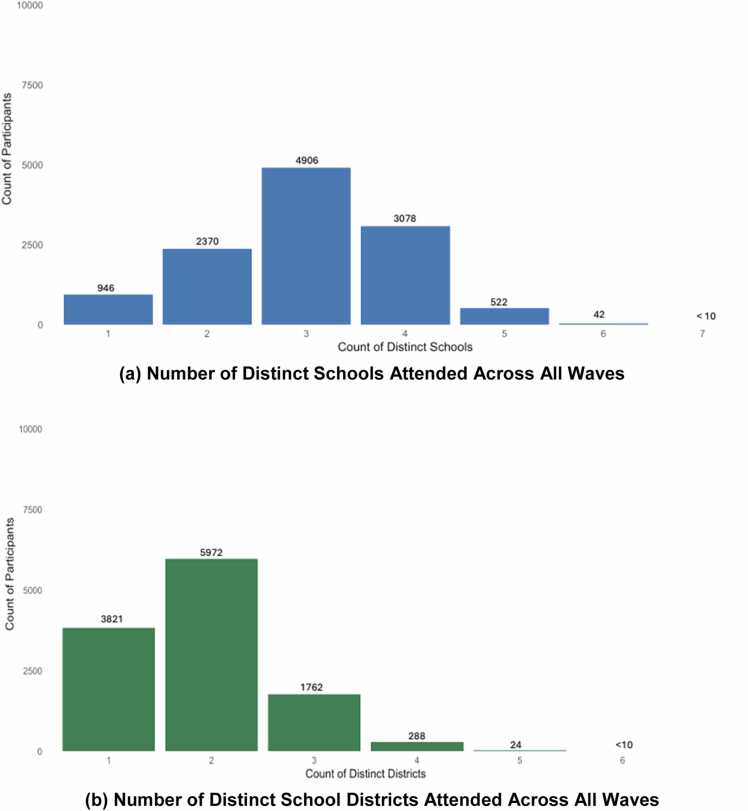
Distribution of School and District Mobility in the ABCD Cohort. **(a) Number of Distinct Schools Attended Across All Waves. (b) Number of Distinct School Districts Attended Across All Waves.***Note.* Panel (a) summarizes the number of distinct schools attended by each participant over all waves, illustrating substantial variability in school mobility (e.g., 4906 participants attended three different schools across seven years). Panel (b) shows the corresponding distribution of district mobility, with most youth attending schools across multiple districts. Together, these distributions highlight the extent of education mobility across early adolescence.

Existing ABCD Study data also offer underutilized opportunities to examine educational contexts as dynamic, multilevel systems. Fig. 2, Fig. 3 highlight substantial movement across schools and districts, including common transitions between elementary, middle, and high school. Despite this variability, relatively few studies have examined how changes in school context influence developmental trajectories, and no studies to date have explicitly modeled school or district clustering using available identifiers. Although ABCD does not currently provide a cumulative count of school changes, annual school identifiers allow researchers to capture whether youth change schools between assessment waves, offering a tractable approach to studying school mobility and transitions. In addition, recent ABCD Study data releases have expanded the availability of anonymized school and district identifiers across participants and timepoints, enabling multilevel analyses that account for clustering within educational contexts. However, the extent to which within-school and district clustering influences observed results remains unclear.

Extending these longitudinal and multilevel opportunities, the School Linked External Data (LED) Workgroup is collaborating with the ABCD Data Analysis, Informatics & Resource Center to link additional waves of SEDA school- and district-level academic achievement data, which is currently included only at baseline. This addition will support examination of how schools and districts performed during and after COVID-related stay-at-home orders. The additional longitudinal data will require restructuring ABCD’s school LED data into a monthly timescale, allowing school-level indicators to vary across time and grade rather than being anchored only at baseline or by other assessment waves. Future studies should also consider the timing of assessments relative to the academic calendar (e.g., weekday versus weekend, school versus summer assessments) and major contextual disruptions such as the COVID-19 pandemic.

### 5.2 Additional educational constructs and pathways

Upcoming ABCD waves will introduce new constructs that expand the ability to examine how educational pathways unfold during late adolescence and emerging adulthood. These additions, including self-reported educational attainment, educational aspirations, and measures of science, technology, engineering, and math (STEM) interests, will enable researchers to capture academic motivation and education-related decision-making at a developmental stage when school choices become increasingly individualized. Incorporating these constructs will be especially important as youth encounter a broader and more diverse set of academic and workforce trajectories.

The ABCD Study consortium is also expanding its efforts to incorporate linked external data for participants who pursue higher education. To support this goal, ABCD has begun collecting the names of the higher education institutions a participant attends, along with the months/years attended. Collecting institutional names and attendance periods will allow future linkage with the Integrated Postsecondary Education Data System (IPEDS), managed by NCES. Linkages include any U.S. institution that receives federal funding and grants degrees or training certificates, such as 2- and 4-year colleges and universities, as well as certificate programs (e.g., cosmetology, massage, welding, culinary school). IPEDS provides comprehensive information on institutional characteristics, such as tuition and fees, student demographics, as well as completion and retention rates. As more external datasets potentially become available, additional linkages may also be possible (e.g., campus-level measures of substance use and safety), which will facilitate richer contextual analyses of higher education experiences in emerging adulthood.

### 5.3 Reframing education attainment beyond traditional metrics

As ABCD participants transition beyond compulsory schooling, the range of postsecondary pathways will expand dramatically. Historically, educational attainment has been operationalized using traditional milestones, such as years of education completed, college enrollment, and degree attainment, reflecting a period when continued schooling often followed a linear path toward in-person two- and four-year college degrees. Indeed, there is robust literature linking involvement in traditional pathways of college and advanced education with economic advantage and better health outcomes (Krueger et al., 2019, Lovenheim and Smith, 2022, Zimmerman et al., 2015). Yet, the contemporary education-workforce landscape is highly diverse. Many young adults pursue alternative pathways, including vocational programs, industry-recognized certifications, online or hybrid degrees, and short-term workforce credentials, that do not fit within conventional metrics but remain highly relevant to economic opportunity and well-being.

Many young adults also engage in informal and self-directed learning outside of traditional educational systems. These can include open online courses from universities, hands-on skill-building platforms for coding, science, and maker projects, educational video channels for focused learning, community-based learning through open-source and creative forums, open textbooks and resource libraries for in-depth study, language-learning tools, open data projects to develop analytical abilities, and informal micro-learning resources for practical everyday skills. These numerous options allow individuals to explore interests and gain real-world competence on their own terms. However, these pathways are rarely captured in standard measures of educational attainment but may play an increasingly important role in shaping knowledge, skills, and long-term outcomes. As such, capturing educational attainment will require broader conceptualization and new approaches to capturing these forms of self-guided learning.

Moreover, national enrollment and completion statistics underscore why research frameworks should not assume college-going as the normative trajectory. Although college application rates have climbed in the last decade, overall enrollment among 18- to 24-year-olds has declined slightly from 41% in 2012–39% in 2022 (NCES, 2024). Obtaining a degree also remains far from universal. Approximately 64% of first-time, full-time students beginning a bachelor’s degree complete it within six years (NCES, 2022), with lower estimates depending on demographic characteristics and institutional type (Tesfamariam et al., 2024). Rising costs, debt concerns, regional workforce needs, and the expansion of alternative postsecondary training may further diversify pathways in the coming decade.

Because the ABCD Study Consortium plans to follow participants into adulthood, it is uniquely equipped to document these transitions, characterize emerging postsecondary trajectories, and examine their implications for health and functioning. Future assessments that capture credentialing, skill-based training, and non-degree learning will be essential for understanding adult role attainment, employment-relevant strengths, and long-term developmental and health outcomes.

## 6 Conclusions

Schools are among the most consequential developmental contexts for children and adolescents, shaping learning, behavior, and brain development in ways that accumulate over time. The ABCD Study findings reviewed here demonstrate that school-related factors were consistently associated with neurodevelopmental, academic, and behavioral outcomes, even after accounting for family socioeconomic characteristics. Findings to date also highlight substantial heterogeneity. Educational environments, opportunities, and supports differ across ABCD Study participants, and these differences may become more pronounced as they progress through adolescence. As ABCD Study participants encounter new academic choices and learning environments, upcoming waves will allow researchers to evaluate how these trajectories diverge, stabilize, or shift over time. The continued integration of linked, geospatial, and multi-informant data will be critical for understanding how changing school experiences relate to functional outcomes later in life. Together, these efforts enable the ABCD Study to advance our understanding of how educational pathways unfold and how learning, training, and workforce preparation shape development across the transition to adulthood.

## CRediT authorship contribution statement

**Sarah M. Lehman:** Writing – review & editing, Data curation. **Jolene Tay:** Writing – review & editing, Data curation. **Shermaine Abad:** Writing – review & editing, Visualization, Data curation. **Christine M. Kaiver:** Writing – review & editing, Data curation. **Bruce D. McCandliss:** Writing – review & editing, Supervision, Conceptualization. **Oliver M. Sawi:** Writing – review & editing. **Elizabeth A. Hoffman:** Writing – review & editing, Writing – original draft, Project administration, Methodology, Investigation, Conceptualization. **Ethan A. Roy:** Writing – review & editing. **Sandra A. Brown:** Writing – review & editing, Project administration, Investigation, Funding acquisition, Conceptualization. **Terry L. Jernigan:** Writing – review & editing, Project administration, Investigation, Funding acquisition, Conceptualization. **Erin L. Thompson:** Writing – review & editing, Writing – original draft, Visualization, Supervision, Project administration, Methodology, Investigation, Funding acquisition, Formal analysis, Data curation, Conceptualization. **Amandine Van Rinsveld:** Writing – review & editing. **Gayathri J. Dowling:** Writing – review & editing, Project administration, Conceptualization. **Marybel R. Gonzalez:** Writing – review & editing, Data curation, Conceptualization.

## Declaration of Competing Interest

The authors declare the following financial interests/personal relationships which may be considered as potential competing interests. Erin L. Thompson reports financial support was provided by National Institutes of Health. Christine Kaiver reports financial support was provided by National Institutes of Health. Sarah Lehman reports financial support was provided by National Institutes of Health. Marybel Gonzalez reports financial support was provided by National Institutes of Health. Sandra Brown reports was provided by National Institutes of Health. Terry Jernigan reports financial support was provided by National Institutes of Health. Jolene Tay reports financial support was provided by National Institutes of Health. Drs. Gayathri Dowling and Elizabeth Hoffman contributed to the interpretation of the data and participated in the preparation, review and approval of the manuscript, consistent with their roles as Science Officers on ABCD grants. Other authors declare that they have no known competing financial interests or personal relationships that could have appeared to influence the work reported in this paper.
