# Supplementary material for: From school climate to brain development: What the ABCD study reveals about the educational context of adolescence
Source: Dev Cogn Neurosci. 2026 May 25;80:101746. doi: 10.1016/j.dcn.2026.101746 (PMC13242015; doi:10.1016/j.dcn.2026.101746)
Supplement: Supplementary file 1 — Supplementary material [file mmc1.docx]

**Supplemental Materials**

**Authors:** Erin L. Thompson, Oliver Sawi, Ethan Roy, Shermaine Abad, Christine M. Kaiver, Sarah M. Lehman, Jolene Tay, Marybel R. Gonzalez, Amandine Van Rinsveld, Gayathri Dowling, Sandra Brown, Terry Jernigan, Bruce D. McCandliss, Elizabeth A. Hoffman

**Table S1.** **Summary of Education-Related and Contextual Constructs, Study Design, and Covariates Across Reviewed ABCD Studies**

| **Citation** | **Education-Related Constructs** | **Other Constructs of Interest** | **Study type** | **Covariates included?** |
| --- | --- | --- | --- | --- |
| **3.1 Supportive School Environments and Adolescent Development** | | | | |
| ***3.1.1 Educational environments contribute unique variation to brain development and are not simply proxies for socioeconomic status*** | | | | |
| *Bates et al., 2025 | School environment; school involvement | Whole-brain cortical thickness ; Caregiver monitoring; family cohesion; prosocial behavior; number of friends; neighborhood safety; puberty stage | Longitudinal (base 🡪 2-year follow-up) | Yes (age, sex, puberty stage, caregiver education) |
| Hong et al., 2021 | Perceived school climate/environment | Trauma exposure; neighborhood safety; family environment; cortical thickness and myelin-surrogate markers; mental health concerns | Cross-sectional (baseline) | Yes (age, sex, site, other constructs of interest) |
| Marotta et al., 2025 | Neighborhood educational resources | ~200 variables of socioeconomic status across behavior, cognition, medical history, nutrition, puberty development, social adjustment, mental health, substance use, life experiences, sleep, socioeconomic status, neighborhood characteristics, caregiver characteristics, medications, demographics; state-level variation | Cross-sectional (baseline) | Yes (Constructs of interest) |
| Meredith et al., 2022 | School environment, involvement, disengagement, neighborhood educational opportunities/resources | Family conflict, parental monitoring, caregiver support, neighborhood crime, walkability, deprivation, pollution, economic opportunities, family values, ethnic identity | Cross-sectional (baseline) | Yes (age, sex, race, ethnicity, household income, caregiver education, site and family IDs) |
| *Petrican et al., 2024 | School engagement | Genetic risk for adult-onset disorders; functional connectivity on n-back task; attentional and interpersonal problems; close friendship; physical activity; nutritional intake | Longitudinal (baseline → 3-year follow-up) | Yes (White youth only, chronological age, delay (in months) between baseline and the 2-year follow-up, biological sex, handedness, serious medical problems, scanner site, and average motion per participant for the n-back task) |
| Rakesh, Zalesky et al., 2023 | School environment | Gray/white matter structure, resting-state functional connectivity (rsFC), cortical thickness and surface area , subcortical volume, CBCL mental health | Cross-sectional (baseline) | Yes (income-to-needs; race/ethnicity; neighborhood income; child intelligence; parent acceptance, head motion; site, family nesting) |
| *Roy, Van Rinsveld, et al., 2024 | SEDA district-level educational opportunity (intercept: average standardized test score for third graders from a given school district relative to the national average; slope: year-to-year growth in standardized test scores for students from a district relative to the national average) | White matter development; family conflict | Longitudinal (baseline → 2-year follow-up) | Yes (age, log-transformed income-to needs ratio, parental education, family cohesion, family conflict, biological sex, pubertal status; nesting of family and site) |
| *Zhou et al., 2025 | COI–Education Opportunity | NIH Toolbox cognition, gray matter volume, cortical thickness | Longitudinal (baseline → 2-year) | Yes (age, sex, and intracranial volume; household material hardship, family conflict, administration method) |
| ***3.1.2 Educational factors also help explain why associations between brain networks and cognitive performance differ across socioeconomic contexts*** | | | | |
| Ellwood-Lowe et al., 2021 | School type; School environment, involvement, disengagement | lateral frontoparietal network (LFPN) – default mode network (DMB) connectivity; poverty status; neighborhood safety | Cross-sectional (baseline) | Yes (age, sex, race, ethnicity, caregiver education, caregiver marital status, intrusive behavior, financial stress, neighborhood crime, parental monitoring) |
| Hackman et al., 2022 | School climate | internalizing and externalizing symptoms; cortical thickness, surface area, subcortical volumes; family income as a moderator | Cross-sectional (baseline) | Yes (age, sex, race/ethnicity, income-to-needs, parental education, site) |
| Rakesh et al., 2021 | School environment | Neighborhood disadvantage (ADI), resting-state functional connectivity, positive parenting, general cognitive ability, executive function, and learning/memory, externalizing, internalizing, and total mental health problems, sex differences | Cross-sectional (baseline) | Yes (sex (in models where sex was not a moderator), age, scanner type, and mean framewise displacement) |
| ***3.1.3 Reading and brain development influence one another over time, with early biological factors shaping these relationships*** | | | | |
| Ahmed et al., 2024 | Receptive vocabulary; oral reading | Maternal hypertensive disorders; cognitive performance, birthweight; body mass index; whole-brain cortical thickness | Cross-sectional (baseline) | Yes (age, sex, race, education, family income, history of traumatic exposure, preterm birth, cesarean delivery, and any birth complications, maternal age at birth, maternal education, maternal substance use during pregnancy and maternal diabetes and anemia during conception, MRI scanner, study site) |
| Byington et al., 2023 | Picture Vocabulary; Oral Reading Recognition | Polyneuro risk scores, distributed functional connectivity patterns, resting-state fMRI networks; fluid cognition | Cross-sectional (baseline) | Yes (site, age, sex, ethnicity, grade, highest level of parental education, handedness, combined family income, and exposure to anesthesia; family ID)) |
| Carrión-Castillo et al., 2023 | Picture Vocabulary; Oral Reading Recognition | Cortical surface area and thickness; polygenic influences | Cross-sectional (baseline) | Yes (age, sex, family income, parental education, family ID, site, scanner) |
| Coccaro et al., 2024 | Picture Vocabulary; Oral Reading Recognition | White matter volume; fractional anisotropy; mean diffusivity; visuospatial processing; fluid intelligence; autism spectrum disorder; non-verbal learning disability; social skills | Cross-sectional (baseline) | None |
| Eckert et al., 2024 | Picture Vocabulary; Oral Reading Recognition | Attention, executive function, episodic memory, processing speed, working memory, surface area auditory cortex asymmetry | Cross-sectional (baseline) | Yes (age, sex, handedness, parental education ,scanner/site) |
| Fekson et al., 2023 | Oral Reading Recognition | Inhibitory control measures; caregiver-reported symptoms of ADHD; resting-state default-mode network functional connectivity profiles | Cross-sectional (baseline) | Yes (age, sex, grade, handedness, race-ethnicity, family income, parental education, motion) |
| Langensee et al., 2023 | Picture Vocabulary; Oral Reading Recognition | Whole-brain grey-matter volume; macrostructural clusters (occipital fusiform, lingual gyrus, cerebellum) | Cross-sectional (baseline) | Yes (age, sex, total GM volume, fluid cognition; site/scanner) |
| *Li, Zhao, et al., 2024 | Reading time; Picture Vocabulary; Oral Reading Recognition | Different types of screen time; brain structure; rule-breaking and aggressive behaviors; internalizing concerns | Longitudinal (baseline → 2-year follow-up) | Yes (age, sex, race, ethnicity, household income, parental education) |
| *Ma et al., 2022 | Receptive vocabulary; oral reading | Gestational age; cortical and subcortical volumes | Longitudinal (baseline → 2-year follow-up) | Yes (Age, sex, body mass index, family income, parental education year, race, puberty score, maternal age at delivery, and prenatal exposure to any kind of substance before or after maternal knowledge of pregnancy) |
| *Menu et al., 2025 | Receptive vocabulary; oral reading ; Caregiver-reported academic grades | Preterm birth; Cognitive performance; Cortical volume, thickness, and area and subcortical volumes; mental health concerns | Longitudinal (baseline → 2-year follow-up) | Yes (sex, age, and site) |
| Owens et al., 2020 | Picture Vocabulary; Oral Reading Recognition | default mode network–dorsal attention network anticorrelation; fluid cognition; caregiver-reported internalizing, externalizing , and attention concerns cognitive flexibility; sleep | Cross-sectional (baseline) | Yes (child’s sex, age, race, and parental education; family and site IDs) |
| Palmer et al., 2021 | Picture Vocabulary; Oral Reading Recognition | Cortical thickness and surface area | Cross-sectional (baseline) | Yes (sex, age, race/ethnicity, household income, highest parental education, fluid and total cognitive domains; scanner, family ID) |
| Rauschecker et al., 2025 | Reading for pleasure (hours/day); Picture Vocabulary; Oral Reading Recognition | TV viewing, fluid cognition, cortical morphology | Cross-sectional (baseline) | Yes (age, sex, ethnicity, genetic ancestry, family ID, parental education, household income, parental marital status, MRI device serial number, and MRI software version) |
| Roy, Richie-Halford et al., 2024 | Oral Reading Recognition | White-matter fractional anisotropy, demographic predictors, SES | Cross-sectional (baseline) | Yes (age, parental income, neighborhood deprivation; school achievement) |
| *Sun et al., 2024 | Reading for pleasure (years; hours/week); Picture Vocabulary; Oral Reading Recognition | NIH Toolbox cognition; caregiver-reported mental health concerns (total); total brain and intracranial volume; cortical areas | Cross-sectional analyses for neurodevelopment (baseline); Longitudinal analyses for reading and cognition (baseline → 2-year follow-up) | Yes (age, sex, BMI, puberty, race/ethnicity, parental education, family income; scanners; family and site IDs) |
| Tomasi & Volkow, 2021 | School grades; Receptive vocabulary; oral reading | Cortical volume and thickness; Fluid and total cognition | Cross-sectional (baseline) | Yes (Sex, Family income, parental education, neighborhood deprivation, family composition, sleep hours, access to alcohol and cigarettes, extracurricular activities, screen use, sex hormone levels head motion, scanner) |
| Wang et al., 2021 | Receptive vocabulary; oral reading | Structural brain networks (diffusion MRI), clique subgraphs, cognitive ability groups | Cross-sectional (baseline) | Yes (age, gender) |
| Zhuo et al., 2022 | Receptive vocabulary; oral reading | Developmental milestones; neurocognitive performance; youth-reported behavioral concerns | Cross-sectional (baseline) | Yes (sex, maternal age at delivery, race/ethnicity, current marital status, highest educational level ever received, residential average household income, maternal prenatal care utilization, maternal psychological problems, maternal substance use before and after awareness of pregnancy, pregnancy complications, delivery or birth outcomes, and breastfeeding ) |
| ***3.1.4 Supportive educational contexts function across multiple levels to buffer the emergence of mental health symptoms and shape responses to adversity*** | | | | |
| *Brieant & Simmons, 2025 | School environment | Caregiver-reported internalizing and externalizing concerns; Family conflict; parental monitoring; parental acceptance; financial hardship; neighborhood safety | Longitudinal (baseline → 3-year follow-up) | Yes (age, sex, site, family ID, as well as all variables of interest) |
| Crumly-Goodwin & Samek, 2024 | School environment | Emotion dysregulation, emotion regulation strategies, family conflict, peer behavior, household income, caregiver and caregiver partner’s education, neighborhood deprivation | Cross-sectional (3-year follow-up) | Yes (sex, race/ethnicity, constructs of interest) |
| *Gonzalez et al., 2025 | SEDA school academic achievement for children in 3^rd^ grade for each school (i.e., intercept) | Risky decision-making | Longitudinal (baseline → 4-year follow-up) | Yes (age, sex at birth, family group, and site identification) |
| Hackman et al., 2022 | School climate | internalizing and externalizing symptoms; cortical thickness, surface area, subcortical volumes; family income as a moderator | Cross-sectional (baseline) | Yes (age, sex, race/ethnicity, income-to-needs, parental education, site) |
| Harris et al., 2025 | Neighborhood Educational Opportunities | Neighborhood social, economic, and health-related opportunities; poverty, internalizing and externalizing concerns, impulsivity, behavioral inhibition and activation, | Cross-sectional (baseline) | Yes (age, sex at birth, parent education and household income, site and family IDs) |
| *Keyin et al., 2025 | School environment; school disengagement | Subthreshold to clinical anxiety, trauma exposure | Longitudinal (baseline → 3-year follow-up) | Yes (age, sex, handedness, family income, psychiatric family histories, parent education, area deprivation index, recent social deprivation, body mass index, screentime, physical activity, sleep disturbances, and substance use),mental health, parents’ multi-group ethnic identity, neighborhood safety, family conﬂict, prosocial behavior, acceptance by parents, and close friends) |
| *Logan & Lewis-de Los Angeles, 2025 | Caring teachers, Liking school; Receptive vocabulary; oral reading | Positive childhood experiences; Adverse childhood experiences; caregiver-reported mental health concerns, cognitive performance | Cross-sectional (2-year follow-up); (Longitudinal (baseline → 2-year follow-up) | Yes (age, sex, race and ethnicity, puberty stage, and family income) |
| *Martínez, Damme et al., 2024 | School environment and involvement | Peer victimization; parental warmth; depression, separation, anxiety, prodromal psychosis, and ADHD, parental warmth | (Longitudinal (baseline → 2-year follow-up) | Yes (baseline mental health concerns, sex, race, age, puberty, presence of a sibling, household income, caregiver education, caregiver self-reported mental health concerns, neighborhood deprivation index) |
| *Park et al., 2024 | School environment; | Polygenic scores; cognitive performance; supportive parenting; neighborhood socioeconomic status; psychotic-like experiences | Longitudinal (baseline → 2-year follow-up) | Yes (sex, age, genetic ancestry, BMI, marital status of the caregiver, and family history of psychiatric disorders , household income, parental education, financial adversity) |
| *Petrican et al., 2024 | School engagement | Genetic risk for adult-onset disorders; functional connectivity on n-back task; attentional and interpersonal problems; close friendship; physical activity; nutritional intake | Longitudinal (baseline → 3-year follow-up) | Yes (White youth only, chronological age, delay (in months) between baseline and the 2-year follow-up, biological sex, handedness, serious medical problems, scanner site, and average motion per participant for the n-back task) |
| Qiu & Liu, 2023 | School environment, involvement, and disengagement | Parental psychopathology and substance use, birth characteristics, polygenic risk scores, brain structural connectivity, family conflict, caregiver-reported internalizing, externalizing, mania, and prodromal symptoms | Cross-sectional (baseline) | Yes (age, gender, ethnicity, family and site IDs) |
| *Smith & Stamoulis, 2023 | Liking school; getting along with teachers, school setting | Empathy, callous-unemotional traits and behaviors, cultural values, family closeness, cohesion, conflict, religious affiliation, ethnic identity, caregiver monitoring, caregiver warmth, family make-up, caregiver history of substance use and mental health concerns, friendship, bullying, perceived unfair treatment, neighborhood safety, internalizing and externalizing concerns, sleep | Longitudinal (baseline → 2-year) | Yes (sex, race/ethnicity, constructs of interest) |
| *Sun et al., 2024 | Reading for pleasure (years; hours/week); Picture Vocabulary; Oral Reading Recognition | NIH Toolbox cognition; caregiver-reported mental health concerns (total); total brain and intracranial volume; cortical areas | Cross-sectional analyses for reading neurodevelopment (baseline); Longitudinal analyses for reading and cognition (baseline → 2-year follow-up) | Yes (age, sex, BMI, puberty, race/ethnicity, parental education, family income; scanners; family and site IDs) |
| Thapaliya et al., 2024 | School environment | Air pollution, population density, area crime, neighborhood safety, household income, family conflict, early life stress, caregiver-reported internalizing concerns, genomic data, heritability | Cross-sectional (baseline); Meta-analysis | Yes (sex, family and site IDs, environmental constructs of interest) |
| Thapaliya et al., 2021 | School environment | Air pollution, population density, area crime, neighborhood safety, household income, family conflict, early life stress, caregiver-reported internalizing concerns, genomic data | Cross-sectional (baseline) | Yes (sex, family and site IDs, environmental constructs of interest) |
| Urbina-Johanson et al., 2025 | School ethnic density; school type (regular vs alternative) | CBCL internalizing/externalizing/thought | Cross-sectional (baseline) | Yes (sex at birth, child age, maternal age, family income-to-needs ratio, household highest level of education; neighborhood deprivation) |
| Vargas et al., 2025 | School environment; COI-Education domain; Receptive vocabulary; oral reading | Neighborhood deprivation, neighborhood safety, neighborhood segregation, neighborhood cohesion, green space, youth-reported mental health concerns, cognition | Cross-sectional (baseline, with one covariate at the 2-year follow-up) | Yes (age, sex, and income-to-needs, neighborhood dimensions, family and site IDs) |
| *Wallace & Conner, 2025 | School climate total score | Youth suicidal thoughts and behaviors, caregiver-reported internalizing and externalizing concerns, substance use, family conflict, parental monitoring, neighborhood safety, family material hardship, ACEs | Longitudinal (baseline → 2-year follow-up) | Yes (age) |
| *Wen et al., 2023 | School support | Self-injurious thoughts and behaviors, cortical areas, FC, behavioral inhibition, p-factor psychopathology, internalizing and externalizing concerns, ADHD, family support | Longitudinal (baseline → 1-year follow-up) | Yes (age, sex, race/ ethnicity, marital status, parental education level, combined household income, scanner and mean motion) |
| *Yang, Tuy et al., 2025 | School climate; school attendance; school academic grades | Neighborhood deprivation and child opportunity, neighborhood safety, peer behavior, parental warmth, parental monitoring, family conflict, caregiver self-reported internalizing and externalizing symptoms, adolescent internalizing and externalizing concerns, prosocial behavior, ACEs, income-to-needs ratio, family material hardship, caregiver education | Longitudinal (baseline → 2-year follow-up) | Yes (age, sex, baseline psychopathology) |
| **3.2 Adverse School Experiences and Developmental Risk Pathways** | | | | |
| ***3.2.1 Adverse school experiences increase risk for mental health concerns through cumulative, bidirectional processes*** | | | | |
| *Brislin, Choi et al., 2024 | School discipline | Arrests, caregiver-reported internalizing and externalizing concerns; delinquent behaviors, neighborhood opportunities/resources; | Longitudinal (ABCD-SD baseline → 2-year follow-up) | Yes (sex, race, constructs of interest) |
| *Brislin et al., 2025 | School environment, involvement, disengagement | Delinquent behavior, prosocial behavior, callous unemotional traits, psychopathy, fearlessness, aggression, emotion regulation, behavioral inhibition and activation, impulsivity, internalizing and externalizing concerns, peer behavior, parenting strategies, family conflict, neighborhood climate and safety | Longitudinal (ABCD-SD baseline → 1-year follow-up) | Yes (Sex, race, ethnicity, parental education, and household income) |
| *Conley et al., 2024 | School environment | Family conflict, neighborhood safety, Caregiver-reported internalizing and externalizing concerns, stressful life events | Longitudinal (baseline → 2-year follow-up) | Yes (household income, site) |
| *Conley, Hernandez et al., 2023 | School environment | Family conflict, neighborhood safety, youth- and caregiver-reported mental health concerns, difficulties with sleep, prosocial behavior, peer interactions, peer behavior, cognitive ability, susceptibility to perceived peer influence | Longitudinal (baseline → 2-year follow-up) | Yes (county-level crime, household income, siblings via random selection of one child per family; some mental health models also accounted for age, sex, informant, and race/ethnicity) |
| Conley, Rapuano et al., 2023 | School environment | Perceived family and neighborhood threat; executive-network activity; n-back performance | Cross-sectional (baseline) | Yes (sex, age, motion, site, externalizing concerns) |
| *Dash et al., 2023 | Neighborhood educational opportunities/resources | Neighborhood social, economic, and health-related opportunities; life stress; externalizing concerns; twin differences; genetics | Longitudinal (baseline → 1-year follow-up) | Yes (age, sex) |
| Fadus et al., 2021 | School discipline; reasons for discipline, special education needs | Race/ethnicity; family structure; externalizing concerns; age; sex; family income; parental education; family conflict | Cross-sectional (baseline) | Yes (predictors of interest, family IDs) |
| *Feinstein et al., 2024 | “I feel safe at school” | Sexual orientation; social problems/teasing; caregiver-reported internalizing and externalizing concerns | Longitudinal (baseline → 2-year follow-up) | Yes (sex, age, race/ethnicity, site and family ID, sampling weights, preceding wave of each outcome of interest) |
| *Hullenaar et al., 2025 | School lockdown exposure, school district-level socioeconomic status | Caregiver-reported internalizing and externalizing concerns | Longitudinal (baseline → 3-year follow-up) | Yes (child grade, sex, race, ethnicity, trauma history, perceived neighborhood crime and safety, baseline mental health concerns, neighborhood resources/opportunities, site) |
| *Kong et al., 2025 | School environment | Prenatal ozone exposure; neighborhood safety; amygdala and hippocampal volumes; cognition; psychotic-like experiences | Longitudinal (baseline → 2-year follow-up) | Yes (Sex, race/ethnicity, age, scanner, total brain volume, total cognition, baseline amygdala and hippocampus volume, baseline psychotic-like experiences, current school and neighborhood environment, PM_2.5_ , NO_2_ , and O_3_,COVID-19 timing) |
| Loso et al., 2023 | School environment | Gender nonconformity; family conflict; behavioral and emotional health total problems | Cross-sectional (1-year follow-up) | Yes (Child age, race, sex at birth, and highest household education, race/ethnicity, child pubertal status) |
| *Martinez, Cai et al., 2024 | Perceived school environment | Family conflict; caregiver depression; peer victimization; hippocampal volume; depressive symptoms | Longitudinal (baseline → 2-year follow-up) | Yes (age, sex, parent education, race, baseline symptoms) |
| Menken et al., 2022 | Academic grades; Picture Vocabulary; Oral Reading Recognition | Peer victimization; NSSI; suicidality; behavioral problems; internalizing and externalizing concerns, cognitive performance, body mass index | Cross-sectional (baseline) | Yes (race, age, sex at birth, caregiver education level, and family income, site and family IDs) |
| Nagata et al., 2021 | Perceived ethnic discrimination from teachers and students | Perceived ethnic discrimination from other adults outside school | Cross-sectional (1-year follow-up) | Yes (sex, race, ethnicity, household income, parental education) |
| *Niu et al., 2025 | School environment, involvement and disengagement | ACEs; caregiver-reported internalizing/externalizing | Longitudinal (baseline → 3-year follow-up) | Yes (age, sex, race and ethnicity, caregiver education, baseline school and mental health constructs) |
| Schiff & Lee, 2023 | Teacher-report externalizing concerns | Youth- and caregiver-reported externalizing concerns; relational and physical aggression, relational and physical victimization, peer behavior, | Cross-sectional (2-year follow-up) | Yes (Girls only; age, race, ethnicity, highest level of parent education, and family income; date of study participation) |
| *Thompson et al., 2025 | School discipline, neighborhood educational opportunities, school type, Teacher-reported externalizing concerns | Aggregated attitudes and beliefs about Black individuals, neighborhood social, economic, environmental, and health-related opportunities/resources | Longitudinal (baseline → 3-year follow-up) | Yes (race/ethnicity, sex at birth, school grade, caregiver education, secondary caregiver status, caregiver-reported externalizing concerns) |
| *Yan et al., 2025 | Teacher discrimination; student discrimination; teacher-reported internalizing, externalizing, attention problems | Other adult discrimination; parental warmth, youth- and caregiver-reported internalizing, externalizing, attention problems | Cross-sectional (1-year follow-up); Longitudinal sensitivity analyses (1-year follow-up → 2-year follow-up) | Yes (adolescent race/ethnicity, sex at birth, age, sexual orientation, family socioeconomic status, and family conflict) |
| *Yang, Tuy et al., 2025 | School climate; school attendance; school academic grades | Neighborhood deprivation and child opportunity, neighborhood safety, peer behavior, parental warmth, parental monitoring, family conflict, caregiver self-reported internalizing and externalizing symptoms, adolescent internalizing and externalizing concerns, prosocial behavior, ACEs, income-to-needs ratio, family material hardship, caregiver education | Longitudinal (baseline → 2-year follow-up) | Yes (age, sex, baseline psychopathology) |
| ***3.2.2 Adverse school experiences appear to accelerate early substance use*** ***initiation rather than escalation*** | | | | |
| *Choi et al., 2025 | School environment; school involvement; school disengagement; neighborhood-level educational opportunities | Alcohol use; Polygenic risk scores; general cognitive ability, impulsivity, prenatal substance exposure, parental history of substance problems, family conflict, parental monitoring, religion, neighborhood resources; structural and task-based cortical and subcortical gray matter volume | Longitudinal (baseline → 4-year follow-up) | Yes (sex, household income, parental education, race/ethnicity, genetic similarities, pubertal status, total cortical and subcortical whole brain volume) |
| *Green et al., 2024 | School involvement; school engagement; school environment; school discipline, special education services, academic grades | Substance use initiation; 420 predictors across physical health, mental health, self and peer substance use, parenting behaviors, neighborhood safety, pubertal hormone levels, neurocognition | Longitudinal (baseline → 3-year follow-up) | Yes (age at baseline, sex, race, ethnicity, parental income, child religious preference, and parent education) |
| Jelsma et al., 2025 | Perceived ethnic discrimination from teachers and students | Perceived ethnic discrimination from other adults outside school; hair toxicology–based and self-reported substance use | Cross-sectional (1-year follow-up or 2-year follow-up) | Yes (age, sex, family immigration status, parental education, parental employment, and family economic hardship, youth religiosity, positive expectancy of substance use, psychopathology, parental monitoring, peer substance use disapproval, and substance availability) |
| Sanchez et al., 2023 | School environment; school involvement; school disengagement | Alcohol expectancies (positive, negative); prosocial behavior, adverse life events, family history of alcohol-related problems, parental monitoring, family conflict, peer behaviors, aggression and victimization, neighborhood safety, familism, language use in the home, | Cross-sectional (2-year follow-up) | Yes (age, sex at birth, sibling participation, family nativity, race, ethnicity, site and family IDs) |
| ***3.2.3 Adverse school experiences are also linked to other health risks, which, in turn, are associated with poorer academic functioning*** | | | | |
| Allen et al., 2022 | Neighborhood educational opportunities/resources | Body mass index, pubertal stage, dietary information, physical activity, developmental milestones, prenatal exposures, neighborhood crime, pollution, neighborhood social and economic indicators | Cross-sectional (Baseline with dietary intake collected at the 1-year follow-up) | Yes (race, ethnicity, parental marital status, education, work status, earnings of primary caregiver before taxes, whether they have a partner, number of people living in the house, and total combined family income, economic hardship, ) |
| Li, Thomas et al., 2024 | Self-reported academic grades; school day schedule; oral reading | Sleep and discrepancies between school and weekend days; BMI | Cross-sectional (2-year follow-up) | Yes (age, sex, ethnicity, household income, and family ID) |
| Raney et al., 2023 | Perceived unfair treatment by teachers and other students due to ethnicity | Binge-eating behaviors, perceived unfair racial treatment; perceived unfair treatment by adults outside of school | Cross-sectional (1-year follow-up) | Yes (age, sex, race/ethnicity, nativity, household income, caregiver education, site) |
| Waltzman et al., 2024 | Academic grades, detentions/suspensions; special education services; Teacher-reported mental health concerns | Traumatic brain or head injury, internalizing, externalizing, anxious-depressed, and total mental health concerns; sleep patterns | Cross-sectional (baseline) | Yes (sex, age ,race/ethnicity, and total family income) |
| ***3.2.4 Remote learning was linked to lower school engagement and adverse mental health outcomes, but these effects were not uniform and were strongly shaped by family conditions.*** | | | | |
| *Gonzalez et al., 2023 | Difficulty completing school remotely during COVID-related school closures | Pre-pandemic psychosocial and financial adversity; pandemic material hardship; disruptions to routines; parental alcohol use; coping strategies; family well-being indices | Longitudinal (pre-pandemic baseline, 1-year, or 2-year follow-up → June 2020 COVID survey) | Yes (non-siblings only, age, sex, race, ethnicity, caregiver reporter, site ID) |
| *Guillaume et al., 2022 | School format (in-person vs remote); time spent on reading, math, science; school enjoyment; parental involvement in school activities | None | Longitudinal (Oct 2020 COVID survey → Mar 2021 COVID survey) | Yes (Race/ethnicity, sex at birth, caregiver education, household income) |
| *Hamatani et al., 2022 | None directly (school closures discussed conceptually, but no school-climate variables analyzed) | Internalizing and externalizing concerns, parental monitoring | Longitudinal (pre-COVID 2-year follow-up → post-onset of COVID 3-year follow-up) | Yes (race/ethnicity, twin or triplets status, sex, annual household income, parental education, site and family IDs) |
| Hunt et al., 2024 | School vs summer months; pre-COVID vs COVID school periods | Fitbit-measured daily steps, sedentary time, physical activity, COVID period indicators | Cross-sectional (2-year follow-up) | Yes (propensity weights, sex, race/ethnicity, household income, age) |
| *Kiss et al., 2022 | School-related predictors: worry about missing school in person; difficulty completing schoolwork; IEP/504 status | Positive affect; internalizing and externalizing concerns; screen time; sleep; family relationship quality; discrimination related to pandemic | Longitudinal (pre-COVID 2-year follow-up → May-August 2020 COVID surveys) | Yes (age, sex, race, ethnicity, number of siblings, research site, caregiver education, and household size) |
| *Kiss et al., 2023 | School vs weekend sleep, summer break | Sleep timing and quality, screen time and type of use | Longitudinal (pre-COVID 2-year follow-up → May 2020-March 2021 COVID surveys) | Yes (age, sex at birth, race, ethnicity, caregiver education, site ID) |
| *Pelham et al., 2021 | School modality (no school / online / in-person or hybrid) | Substance use; COVID-related financial impact, worry and stress, parents’ self-reported substance use | Longitudinal (pre-COVID semiannual assessments in 2018, 2019, 2020 → May-August 2020 COVID surveys) | Yes (sex, race/ethnicity, caregiver married status, caregiver education, pre-pandemic internalizing and externalizing concerns and parents’ prior substance use) |
| *Raney et al., 2024 | School modality, parental engagement with schoolwork | Positive affect, stress, ACEs, coping behaviors | Longitudinal (pre-COVID baseline and 1-year follow-up → March 2021 COVID survey) | Yes (age, sex, race/ethnicity, caregiver education, household income, pre-pandemic externalizing and externalizing behaviors) |
| *Rosenthal et al., 2022 | Pre-pandemic school environment and involvement; school modality, perceived preparation for next school year, perceptions of remote learning | ADHD symptoms, medication for ADHD, parental monitoring, COVID-related stress and behaviors, sleep, COVID-19 health symptoms | Longitudinal (pre-COVID 1-year follow-up → March 2021 COVID survey) | Yes (race, sex, age matching between ADHD group and non-ADHD control group) |
| *Stinson et al., 2021 | School modality | ACEs, internalizing concerns, COVID distress, positive affect, racial discrimination related to COVID-19, fear of illness | Longitudinal (pre-COVID baseline and 1-year follow-up → May-August 2020 COVID survey) | Yes (sex at birth, race, ethnicity, caregiver education, household income, age, family job/wage loss due to COVID, baseline internalizing, site ID) |
| Xiao et al., 2023 | COVID-related school disruptions | State- and county-level covid policies, financial disruptions due to COVID impact, sadness, stress, positive affect, worry, sleep | Longitudinal  (May 2020 COVID survey → December 2020 COVID survey) | Yes (age, biological sex, race or ethnicity, parental education, parental marital status, household income, county-level COVID incidence, COVID-related news exposure) |
| *Yip et al., 2022 | Remote learning, time spent on schoolwork, difficulty completing schoolwork, perceived preparation for next school year | 1700 pre-pandemic variables, including race, ethnicity, language, family structure, socioeconomic status, and the perceived experience of racism; 289 COVID-related variables including hygiene, discussions around COVID, isolation, financial worry | Longitudinal (pre-COVID baseline → May-August 2020 COVID surveys) | Yes (constructs of interest) |
| ***3.3* Behavioral Health and Subsequent Educational Outcomes** | | | | |
| ***3.3.1 Brain development plays an important role in academic performance, with associations varying across socioeconomic contexts*** | | | | |
| Alnæs et al., 2020 | Parent-reported academic grades in school; School climate | Socioeconomic status; genetics; sleep; religiosity; family structure; cortical morphology, urbanicity, air pollution | Cross-sectional (baseline) | Yes (sex, age, race/ethnicity, siblings, twins, sites, scanners, head motion) |
| *Devakonda et al., 2024 | School disengagement | Caregivers’ familism values; neural reward anticipation | Longitudinal (baseline → 1-year follow-up) | Yes (baseline school disengagement, age, biological sex, parents’ educational attainment, household financial adversity, parents’ gender and nativity) |
| *Ellwood-Lowe et al., 2021 | School type; School environment, involvement, disengagement | lateral frontoparietal network (LFPN) – default mode network (DMB) connectivity; poverty status; neighborhood safety | Cross-sectional (baseline) | Yes (age, sex, race, ethnicity, caregiver education, caregiver marital status, intrusive behavior, financial stress, neighborhood crime, parental monitoring) |
| *Elton et al., 2025 | Youth-reported grades | Adverse life events; functional connectivity changes; internalizing and externalizing symptoms | Longitudinal (baseline → 2-year follow-up) | Yes (propensity weighting on 390 covariates, age, sex, race, ethnicity, puberty scores, family history of depression and substance use, caregiver education, family income, one versus separate homes, scanner, head motion) |
| Tomasi & Volkow, 2021 | School grades; Receptive vocabulary; oral reading | Cortical volume and thickness; Fluid and total cognition | Cross-sectional (baseline) | Yes (Sex, Family income, parental education, neighborhood deprivation, family composition, sleep hours, access to alcohol and cigarettes, extracurricular activities, screen use, sex hormone levels head motion, scanner) |
| *Langensee et al., 2024 | Academic grades | Socioeconomic status: Income-to-needs ratio; neighborhood deprivation; caregiver educations | Longitudinal  (2-year follow-up → 3-year follow-up) | Yes (time, cognitive performance) |
| *Li et al., 2025 | Caregiver-reported academic grades | Greenspace exposure; total surface area, cortical volume, cortical thickness, internalizing, attention, and externalizing problems | Longitudinal (baseline → 2-year follow-up) | Yes (sex, age, income-to-needs ratio, area deprivation index, scanner, family ID, site) |
| *Rakesh et al., 2025 | Youth- and caregiver-reported academic grades | income-to-needs ratio, parental educational attainment, and neighborhood SES via Area Deprivation Index, resting-state functional connectivity | Longitudinal (baseline → 3-year follow-up) | Yes (mean framewise displacement, change in participant age between T1 and T2, age at T2, and sex, race/ethnicity, scanner, family ID) |
| *Yang et al., 2023 | Academic achievement (i.e., caregiver-reported grades); crystallized intelligence (reading and vocabulary) | Sleep timing, sleep duration, resting-state functional connectivity (rs-FC) of cortical networks | Longitudinal (2-year → 3-year follow-up) | Yes (age in months, sex at birth, the interaction between age and sex, race, and study site, parent education level and household income, pubertal status, BMI, sleep duration, and total sleep disturbance) |
| *Yang, Zhou et al., 2025 | Academic achievement (i.e., caregiver-reported grades) | Frontoparietal-striatum connectivity, parental warmth | Longitudinal (baseline → 2-year) | Yes (demographics: age, biological sex, race, and family income) |
| *Zhou et al., 2025 | COI–Education Opportunity | NIH Toolbox cognition, gray matter volume, cortical thickness | Longitudinal (baseline → 2-year) | Yes (age, sex, and intracranial volume; household material hardship, family conflict, administration method) |
| ***3.3.2 Modifiable school structures, such as start times and daily schedules, shape sleep, and, in turn, academic outcomes*** | | | | |
| Li, Thomas et al., 2024 | Self-reported academic grades; school day schedule; oral reading | Sleep and discrepancies between school and weekend days; BMI | Cross-sectional (2-year follow-up) | Yes (age, sex, ethnicity, household income, and family ID) |
| *Wallace et al., 2025 | School environment | Caffeine intake, screen time, stress, peer behavior, sleep patterns, anxiety and depression, perceived stress, | Longitudinal (baseline → 4-year follow-up) | Yes (age, sex, household income, Hispanic ethnicity, family ID) |
| *Wang et al., 2025 | School-night sleep duration; school climate | Self-reported and Fitbit sleep patterns, perceived unfair treatment, substance use intention | Longitudinal  (1-year follow-up → 3-year follow-up) | Yes (race, ethnicity, sexual orientation, body mass index, age, sex at birth, gender congruence, caregiver education, employment status, and economic hardship, generational status, bedtime preference, religiosity, psychopathology) |
| *Yang et al., 2023 | Academic achievement (i.e., caregiver-reported grades); crystallized intelligence (reading and vocabulary) | Sleep timing, sleep duration, resting-state functional connectivity (rs-FC) of cortical networks | Longitudinal (2-year → 3-year follow-up) | Yes (age in months, sex at birth, the interaction between age and sex, race, and study site, parent education level and household income, pubertal status, BMI, sleep duration, and total sleep disturbance) |
| Yip et al., 2025 | School start times | Self-reported and Fitbit sleep patterns, | Cross-sectional (2-year follow-up but with three-week actigraphy) | Yes (race, ethnicity, age, sex, generational status, pubertal status, chronotype, body mass index, perceived racial and ethnic unfair treatment, caregiver education, caregiver employment status, family economic hardship, family structure, neighborhood deprivation, site ID) |
| *Zhang et al., 2025 | School environment, school involvement and school disengagement; academic grades | Fitbit sleep and step counts, seasonal timing/daylength, internalizing and externalizing concerns, prodromal psychosis, substance and caffeine use, resting state functional connectivity, cortical thickness, subcortical grey matter volumetric measures, fractional anisotropy | Longitudinal  (2-year follow-up → 4-year follow-up) | Yes (caregiver marital status, parental highest education, family income, parents’ emotional, behavioral and drug use problems, neighborhood safety, family conflict, scanner type, head motion) |
| ***3.3.3 Screen use may reflect broader patterns of disengagement and contextual disadvantage rather than serving as a primary driver of academic outcomes*** | | | | |
| *Li, Zhao, et al., 2024 | Reading time; Picture Vocabulary; Oral Reading Recognition | Different types of screen time; brain structure; rule-breaking and aggressive behaviors; internalizing concerns | Longitudinal (baseline → 2-year follow-up) | Yes (age, sex, race, ethnicity, household income, parental education) |
| Paulich et al., 2021 | Academic grades; School day versus weekend screen use | Screen use type; internalizing, externalizing, and attention concerns, sleep, friendships | Cross-sectional (baseline) | Yes (sex, household income, race/ethnicity) |
| *Shao et al., 2023 | Perceived racial/ethnic unfair treatment by teachers and other students | Screen time | Longitudinal (Unknown) | No but examined profile differences across sex, race/ethnicity, caregiver education, perceived racism |
| *Xu et al., 2025 | School environment, involvement, disengagement | Screen use, internalizing, externalizing, attention, social, somatic, and thought problems, impulsivity and behavioral inhibition/activation, family conflict, peer behavior | Longitudinal  (2-year follow-up → 4-year follow-up) | Yes (age, race, ethnicity, parental marital status, parental highest education, and family income) |
| **3.4 Methodological and Developmental Considerations** | | | | |
| ***3.4.1 The ABCD Study’s multi-informant and multimethod design provides a unique opportunity to capture different dimensions of adolescents’ school experiences and functioning*** | | | | |
| Cordova et al., 2022 | Teacher-rated ADHD | Caregiver-rated ADHD; comorbid psychiatric disorders (Mood Disorders, Disruptive Behavior Disorders, and Anxiety and Fear Disorders), Polygenic scores, cognition | Cross-sectional (baseline) | Yes (propensity weights, sex, age, family and site nesting) |
| *Marek et al., 2025 | Parent-reported academic grades | Self-regulation factors: urgency, premeditation, perseverance, sensation seeking, impulsivity, inhibition, fun seeking, drive, reward responsiveness; cognitive control, cognitive flexibility, working memory, attentional concerns, | Longitudinal (baseline → 2-year follow-up) | Yes (baseline grades, one participant per family) |
| Pintos Lobo et al., 2025 | Academic grades and special services received in school | ADHD, internalizing and externalizing concerns, prosocial behavior, social responsiveness, family conflict, peer relationships, resting-state functional connectivity | Cross-sectional (baseline) | Yes (age, sex, race/ethnicity, pubertal status, caregiver education, household income, neighborhood disadvantage, family, site, medication use, and comorbidity of anxiety disorder(s), conduct disorder, and/or oppositional defiant disorder) |
| Rohr et al., 2025 | Weekday vs weekend sleep timing (school schedule context) | Self-reported and Fitbit sleep patterns, body mass index | Cross-sectional (2-year follow-up but with three-week actigraphy) | Yes (age, sex, household income, pre/post-COVID sensitivity analysis |
| Schiff & Lee, 2023 | Teacher-report externalizing concerns | Youth- and caregiver-reported externalizing concerns; relational and physical aggression, relational and physical victimization, peer behavior, | Cross-sectional (2-year follow-up) | Yes (Girls only; age, race, ethnicity, highest level of parent education, and family income; date of study participation) |
| Tang et al., 2025 | School victimization; school climate | Other victimization types; Internalizing and externalizing concerns; parental monitoring, parental acceptance, neighborhood safety | Cross-sectional (ABCD-SD baseline) | Yes (child gender, child race and ethnicity, child age, interview year, and total estimated family income) |
| *Wang et al., 2025 | School-night sleep duration; school climate | Self-reported and Fitbit sleep patterns, perceived unfair treatment, substance use intention | Longitudinal  (1-year follow-up → 3-year follow-up) | Yes (race, ethnicity, sexual orientation, body mass index, age, sex at birth, gender congruence, caregiver education, employment status, and economic hardship, generational status, bedtime preference, religiosity, psychopathology) |
| Watts et al., 2022 | Teacher- reported internalizing, externalizing, and attention concerns | Mental health concerns from youth and caregiver reports; | Cross-sectional (6-month follow-up) | None (excluding constructs of interest) |
| *Weigard et al., 2023 | Teacher-reported ADHD symptoms; neighborhood-level educational quality and opportunity | Caregiver-reported ADHD, Neurocognition, screen time, impulsivity, parental monitoring, family conflict, their school system, and neighborhood crime | Longitudinal (baseline → 2-year follow-up) | Yes (one sibling per family, age, sex, race, ethnicity, parental marital status, parental income, and parental education, neighborhood poverty, BMI, site) |
| Zhao et al., 2025 | Receptive vocabulary; oral reading | Screen use (self-report and EARS), internalizing and externalizing concerns, other cognitive domains | Cross-sectional (4-year follow-up with smartphone tracking across three weeks) | Yes (age, sex, race/ethnicity, family income, parental education, parental marital status, pubertal development, family id) |
| ***3.4.2 The timing of assessment relative to the school context may influence observed neurodevelopmental patterns*** | | | | |
| Hu et al., 2023 | Time-of-week (school day vs weekend); time-of-year (school year vs summer vacation) | Scan time-of-day; resting-state whole-brain, network-specific and regional topological properties; cognitive task performance | Cross-sectional (baseline) | Yes (age, sex, race, ethnicity, family income, BMI, screen time, physical activity, propensity weights) |

*Note.* Studies may be repeated as they could be included in multiple domains. Longitudinal data could have also included intermediary waves.

ABCD-SD = Adolescent Brain Cognitive Development Substudy. ADHD = Attention-deficit/hyperactivity disorder. SEDA = Stanford Education Data Archive.

* = Denotes longitudinal study.
